# EpCAM promotes endosomal modulation of the cortical RhoA zone for epithelial organization

**DOI:** 10.1038/s41467-021-22482-9

**Published:** 2021-04-13

**Authors:** Cécile Gaston, Simon De Beco, Bryant Doss, Meng Pan, Estelle Gauquelin, Joseph D’Alessandro, Chwee Teck Lim, Benoit Ladoux, Delphine Delacour

**Affiliations:** 1 Cell Adhesion and Mechanics, Institut Jacques Monod, CNRS UMR7592, Paris Diderot University, Paris, France; 2 Mechanobiology Institute, T-lab, Singapore, Singapore

**Keywords:** Cell biology, Molecular biology

## Abstract

At the basis of cell shape and behavior, the organization of actomyosin and its ability to generate forces are widely studied. However, the precise regulation of this contractile network in space and time is unclear. Here, we study the role of the epithelial-specific protein EpCAM, a contractility modulator, in cell shape and motility. We show that EpCAM is required for stress fiber generation and front-rear polarity acquisition at the single cell level. In fact, EpCAM participates in the remodeling of a transient zone of active RhoA at the cortex of spreading epithelial cells. EpCAM and RhoA route together through the Rab35/EHD1 fast recycling pathway. This endosomal pathway spatially organizes GTP-RhoA to fine tune the activity of actomyosin resulting in polarized cell shape and development of intracellular stiffness and traction forces. Impairment of GTP-RhoA endosomal trafficking either by silencing EpCAM or by expressing Rab35/EHD1 mutants prevents proper myosin-II activity, stress fiber formation and ultimately cell polarization. Collectively, this work shows that the coupling between co-trafficking of EpCAM and RhoA, and actomyosin rearrangement is pivotal for cell spreading, and advances our understanding of how biochemical and mechanical properties promote cell plasticity.

## Abstract (web summary)

The organization and force-generating property of actomyosin dictate the plasticity and behaviour of cells but the spatio-temporal regulation of this network is unclear. Here, the authors show that coupling between EpCAM/RhoA co-trafficking and actomyosin rearrangement is pivotal during cell spreading and polarization.

## Introduction

Biological processes such as cell division, extrusion, maintenance of cell shape, or morphogenetic movements rely in particular on the regulation of the patterning and activity of cell contractility^1–9^. A growing number of studies are investigating the effects of the coupling between biochemical and mechanical stimuli on cell shape and behavior^9^. Stress fibers (SFs) are actin-based structures where this coupling is easily accessible, as they generate and/or transmit forces (i.e., mechanical input) and are connected to biochemical signaling hubs, such as focal adhesions (FAs)^10,11^.

Three subtypes of SFs have been previously described^12^. Ventral stress fibers (VSFs) are formed by cross-linked actin bundles with alternating patterns of α-actinin and myosin-IIA. VSFs are on each side attached to FAs and constitute the force-generating fibers^13^. Radial fibers (RFs), also known as dorsal fibers, elongate perpendicularly from a FA present at the cell periphery by a formin-dependent actin polymerization process^14^. As they are devoid of myosin-IIA, RFs transmit forces only passively, from the circumferential arcs (CAs) to which they are anchored on the dorsal side of the cell to the FA from which they grow^15^. Thus, RFs exert only low forces on the substrate^16,17^. Moreover, the actin polymerization at the leading edge drives the movement of F-actin toward the cell center in a process called retrograde flow^18^. CAs, also called transverse arcs, arise from the bundling of short actin and myosin-II filaments as a result of actin retrograde flow near the dorsal side of the cell^12,14^. Hotulainen and Lappalainen first reported that VSFs arise from the fusion of RFs with their associated CAs following the arc contraction^14^. This process, called SF maturation, was described at the molecular level as a myosin-IIA-dependent process. Contractility blocks further RF growth and promotes their maintenance at the extremity of CAs^19^. The incorporation of myosin-IIA into SFs during their maturation depends on the dynamic properties of the actin-binding protein α4-actinin^20,21^. Other studies have described the existence of de novo SF assembly based on the concatenation of short actin filaments^22,23^. SF formation and dynamics are often used as markers of symmetry breaking and acquisition of front–rear polarity^24,25^.

Myosin-II’s ability to generate forces depends on the phosphorylation of its regulatory light chain (MRLC). Mono-phosphorylation on serine 19 (S19) is sufficient to increase myosin-IIA activity. In addition, di-phosphorylation (T18/S19) favors myosin-IIA assembly and stability on the actin filaments^26–28^. Of note, fluorescence recovery after photobleaching experiments performed on mutant constitutive mono- (S19D) or di-phosphorylated (T18D/S19D) forms of myosin-IIA have shown that dephosphorylation is required for the motor protein to move along actin fibers^27^. Several signaling pathways modulate myosin-II activity, among which the Rho pathway has been extensively studied^29–31^. Rho-associated kinases (ROCKs) promote contractility not only by phosphorylating MRLC but also by inactivating the myosin light chain phosphatase (MLCP)^32,33^. Upstream, the small GTPase RhoA mediates the stimulation of ROCK^34^. Therefore, RhoA distribution and level of activity must be finely controlled to ensure a correct contractile response in the cell^35^. Rho GTPases are described as molecular switches, as they move between activated (GTP-loaded) and inactivated (GDP-loaded) states. This cycling between inactive and active form is important to maintain active RhoA zones and would take place near the plasma membrane^31,36,37^. It is now clear that the inactivation of RhoA is just as important as its activation. While the activation of RhoA facilitates its translocation to the plasma membrane (Michaelson et al.^38^), its inactivation enables a pulsatile behavior of myosin-II activity, needed for efficient contractility^39,40^. Current research efforts are particularly focused on understanding the upstream mechanisms controlling this canonical regulator of contractility.

While cell divisions, extrusions, and rearrangements occur continuously in epithelia, the integrity of tissues must nevertheless be preserved. To this end, the actomyosin activity and its tension levels are carefully monitored at the level of adherent and tight junctions^41,42^. The destabilization of the actin cytoskeleton at the cell contacts prevents the proper recruitment of E-cadherin, the main epithelial cadherin, and leads to defective adherent junctions^3,43^. Similarly, tight junctions contribute to tension homeostasis as they are anchored to the actin cytoskeleton^44^. Tricellulin, a component of tricellular tight junctions, promotes actin polymerization mediated by Cdc42 and thus regulates junctional tension and cell shape in epithelial monolayers^45^. A crosstalk also occurs between the different cell junctions. For example, ZO-1, a tight junction component, participates in the regulation of the tension exerted on neighboring adherent junctions in Xenopus embryos^46^.

EpCAM (Epithelial Cell Adhesion Molecule) is a transmembrane protein that is only expressed in epithelial cells under physiological conditions. EpCAM was originally described as a Ca^2+^-independent cell–cell adhesion molecule essential for epithelial integrity^47,48^. Indeed, during early stages of zebrafish or Xenopus development, *EPCAM* knockout (KO) leads to epiboly defects and numerous lesions of the future epidermis (Slanchev et al. 2009^49^; Maghzal et al.^50^). In addition, the loss of EpCAM causes the development of a rare human disease called Congenital Tufting Enteropathy (CTE). CTE is characterized by the formation of distinctive lesions in the intestinal epithelium^51–53^. These studies point toward a role of EpCAM in epithelial maintenance; however, the mechanisms involved remain unclear. The expression of EpCAM limits the formation of cell junctions by E-cadherin^54–56^. In mice, *EPCAM*-KO also leads to the loss of Claudin-7, a component of tight junctions^57^. By complexing with Claudin-7, EpCAM loses its ability to oligomerize with other EpCAM proteins, without impacting cell–cell adhesion^58^. In light of these results, the fact that EpCAM, as an adhesion molecule, was necessary for epithelial integrity was puzzling. Recent structural analyses of EpCAM extracellular domain demonstrated that its stated function as a homophilic adhesion molecule needs to be revised^59,60^. In addition, a link between EpCAM and the actin cytoskeleton was proposed in the 1990s. Knockdown (KD) or overexpression of EpCAM influenced actin organization^61^, but the mechanism involved in the actin network remodeling remained unclear. More recently, several reports highlighted that EpCAM acts as a regulator of actomyosin contractility in epithelial assemblies. The impact of EpCAM on contractility modulation was revealed by Fagotto and colleagues. They showed that *EPCAM* KD in Xenopus embryos resulted in increased cell contractility and internalization of C-cadherin (Maghzal et al.^50,62^). They also confirmed these results in human Caco2 cells. This cell line exhibits spontaneous terminal differentiation in enterocytes when reaching confluency, a property that is frequently used to mimic the intestinal epithelium or to study apico-basal epithelial polarity^63,64^. Moreover, the loss of EpCAM triggers an inappropriate distribution and magnitude of actomyosin activity at tricellular contacts. This defect impacts apico-basal epithelial polarity and overall monolayer arrangement in CTE patients and Caco2 cells^53,65^. In their work, Fagotto and colleagues showed that excess of cell contractility in mutant Xenopus explants was under the control of nPKC-dependent extracellular signal-regulated kinase (Erk) signaling (Maghzal et al.^50,62^). However, the Erk signaling pathway is not the only mechanism involved. In fact, in the same studies, treatments decreasing RhoA signaling were just as effective as treatments with PKCη inhibitor in restoring a normal phenotype in morpholino-treated (*EPCAM*-MO) explants. Although the expression of a dominant-negative form of RhoA was even more effective than that of a dominant-negative protein kinase C (PKC) in limiting the loss of integrity in *EPCAM*-MO explants^50^, the authors did not further explore RhoA-dependent pathways linked to EpCAM activity. Given these results and the numerous feedback controls between the different pathways that regulate contractility, the molecular mechanism that couple EpCAM to cell contractility deserves further analyses.

In this work, we analyze isolated epithelial cells to understand the direct impact of EpCAM on actin cytoskeleton organization and actomyosin-based contractility. Here, we show that EpCAM participates in the modulation of RhoA signaling and controls the development of contractility for SF maturation and single-cell polarity. Moreover, we reveal that EpCAM is needed for endosomal remodeling of the active RhoA zone at the cell cortex during cell spreading and polarization.

## Results

### EpCAM is required for cell polarization independently of cell–cell contacts

**Fig. 1:**
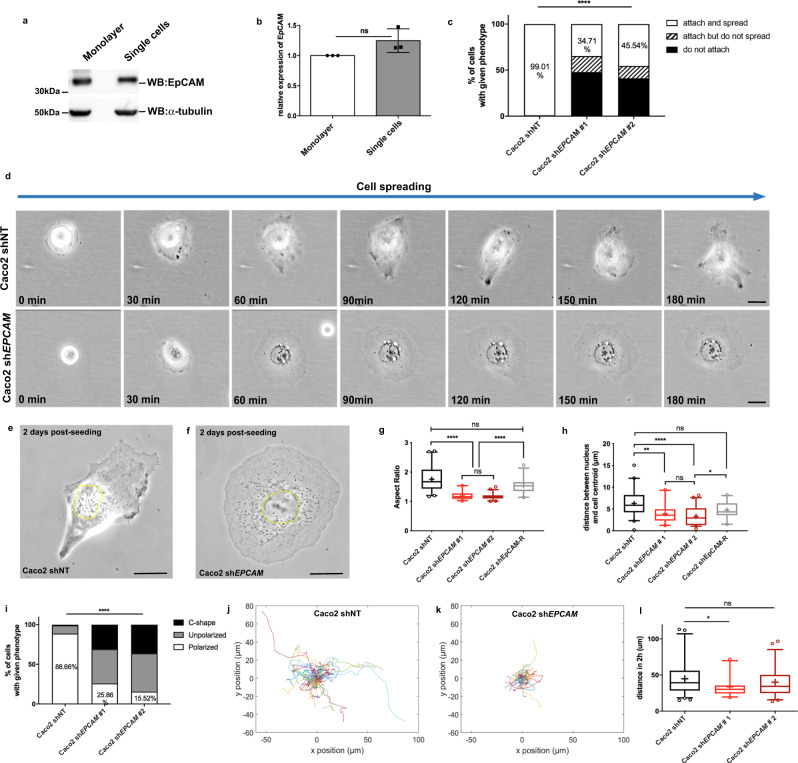
EpCAM is required for single-cell front–rear polarization and migration. **a**, **b** Western blot analysis (**a**) and statistical analysis (**b**) of EpCAM expression in Caco2 21-day monolayers or single cells spread on collagen-coated petri dish and coverslips, respectively. α-Tubulin was used as a loading control. Unpaired *t* test with Welch’s correction, *p* = 0.1602 (non-significant). Data are mean ± SD. **c** Statistical analysis of cell adhesion within 3 h post-seeding for control (Caco2 shNT) or *EPCAM*-KD (Caco2 sh*EPCAM* #1 and #2) cells. The percentage of cells that attach and spread, attach but do not spread, and cells that do not attach is represented. The exact number of cells (and %) in each category is available in Supplementary Table 2. *N* (shNT) = 101 cells, *N* (sh*EPCAM* #1) = 121, *N* (sh*EPCAM* #2) = 112. Chi-square test computed on the number of cells with given phenotype indicates a *p* value < 0.0001. **d** Phase-contrast time lapse of control and *EPCAM*-KD cells during cell spreading. Imaging was performed for 3 h right after seeding. Scale bar, 5 μm. **e**, **f** Phase-contrast representative images of control (**e**) or *EPCAM*-KD (**f**) 2-day post-seeding single cells. Scale bar, 5 μm. **g** Statistical analysis of the aspect ratio in control or *EPCAM*-KD cells. Aspect ratio for shNT cells = 1.756 ± 0.42 (mean ± SD), sh*EPCAM*#1 = 1.188 ± 0.13, sh*EPCAM*#2 = 1.17 4 ± 0.11, sh*EPCAM*-R = 1.537 ± 0.25. The whiskers of the box plot represent the 5–95 percentile confidence intervals; the mean is displayed as a cross. *N* (shNT) = 41 cells, *N* (sh*EPCAM*#1) = 33, *N* (sh*EPCAM*#2) = 44, *N* (sh*EPCAM*-R) = 30. Kruskal–Wallis test and Dunn’s multiple comparison tests, ****p < 0.0001. **h** Statistical analysis of the distance between the nucleus and the centroid in control or *EPCAM*-KD cells. Distance between the nucleus and the centroid in shNT cells = 6.28 ± 2.96 (mean ± SD), sh*EPCAM*#1 = 3.911 ± 2.18, sh*EPCAM*#2 = 3.321 ± 2.15, sh*EPCAM*-R = 4.769 ± 1.88. The whiskers of the box plot represent the 5–95 percentile confidence intervals; the mean is displayed as a cross. *N* (shNT) = 41 cells, *N* (sh*EPCAM*#1) = 33, *N* (sh*EPCAM* #2) = 45. Kruskal–Wallis test with Dunn’s multiple comparison test, *adjusted *p* = 0.0212, **adjusted *p* = 0.0014, ****adjusted *p* < 0.0001. **i** Statistical analysis of polarity phenotypes in control or *EPCAM*-KD cells. The percentage of cells polarized, unpolarized, and C-shaped is represented. The exact number of cells (and %) in each category is available in Supplementary Table 3. *N* (shNT) = 97 cells, *N* (sh*EPCAM*#1) = 58, *N* (sh*EPCAM* #2) = 58. Chi-square test computed on the number of cells with given phenotype indicates a *p* value < 0.0001. **j**, **k** Color map of cell tracks in control (**j**) and *EPCAM*-KD (**k**) cells. **l** Statistical analysis of the distance traveled by control and *EPCAM*-KD cells within 2 h. The whiskers of the box plot represent the 5–95 percentile confidence intervals; the mean is displayed as a cross. *N* (shNT) = 67 cells, *N* (sh*EPCAM*#1) = 37, *N* (sh*EPCAM* #2) = 52. Kruskal–Wallis test and Dunn’s multiple comparison test, *p = 0.0481. Each experiment was replicated independently three times. ns non-significant.

In an epithelial cell context, EpCAM has been exclusively studied in cell clusters and monolayers^66–68^. But recent studies suggested that EpCAM may in fact not be a homophilic cell adhesion molecule^60^. We and others have previously shown that EpCAM impacted the actomyosin network, and we hypothesized that this function may be independent of cell–cell junctions. To investigate this idea, we first tested whether EpCAM was present in significant amount in single cells. Caco2 cells were cultured on collagen-coated substrates either for 21 days to obtain polarized monolayers or for 2 days at very low density to obtain a vast majority of single cells. We observed that EpCAM was already expressed in single cells at a level comparable to that of monolayers (Fig. 1a, b). This suggested that EpCAM could play important functions in culture very early, at the single-cell stage, before the establishment of a confluent and polarized monolayer. To determine the impact of *EPCAM* KD on the behavior and organization of individual cells, we used the previously established stable Caco2 control or *EPCAM* KD (*EPCAM*-KD) clones^53^ and assessed cell spreading and migration by time-lapse imaging. Directly after seeding, almost all of the control cells were able to attach and spread while half of the *EPCAM*-KD cells were not (Fig. 1c). Within 2 h, the control cells had completed their spreading and spontaneously developed an elongated, polarized shape before crawling actively (Supplementary Movie 1 and Fig. 1d, e). In contrast, mutant cells spread abnormally and failed to polarize (Supplementary Movie 2 and Fig. 1d). Their isotropic circular or fried-egg shape, characteristic of single non-polarized cells, was stable over 2 days post-seeding (Fig. 1f). Quantification of the aspect ratio (Fig. 1g) and the distance between the nucleus and the centroid of the cell (Fig. 1h) further confirmed the polarization defect induced by the loss of EpCAM (aspect ratio of 1.76 + /− 0.42 for control cells; 1.19 + /− 0.13 and 1.17 + /− 0.11 for *EPCAM* shRNA#1 and #2 (mean + /− SD)). We used a short hairpin RNA (shRNA)-resistant EpCAM-GFP construct to validate the specificity of these defects. The aspect ratio and the polarization of the nucleus were recovered with the EpCAM rescue (aspect ratio of 1.54 + /− 0.25; Fig. 1g, h). Noteworthy, a subpopulation of the fried-egg-shaped *EPCAM*-KD cells underwent a symmetry breaking event and eventually displayed a large C-shaped protrusion, reminiscent of fish keratocyte migration mode (31 and 36% in Caco2 sh*EPCAM*#1 and #2, respectively; Fig. 1i and Supplementary Fig. 1). A similar phenotype has been reported after inhibition of myosin-II activity in fibroblasts^69^. Nevertheless, the C-shaped *EPCAM*-KD cells did not exhibit directional motility but instead spun around and rapidly detached from the substrate (Supplementary Movie 3). The loss of front–rear polarity in mutant cells prompted us to test their migratory behavior. Whereas the control cells exhibited active motile behavior, as evidenced by the measurement of cell displacement (Fig. 1j and Supplementary Movie 1), *EPCAM*-KD cells motility on the substrate was less extensive (Fig. 1k, l and Supplementary Movie 2). Taken together, the data showed that: (i) EpCAM plays a role in single cells, independently of cell–cell contacts, and (ii) the absence of EpCAM causes changes early in the acquisition of cell morphology, generating a stable unpolarized state that impinges on the migratory behavior of epithelial cells.

### Block of SF maturation in the absence of EpCAM

**Fig. 2:**
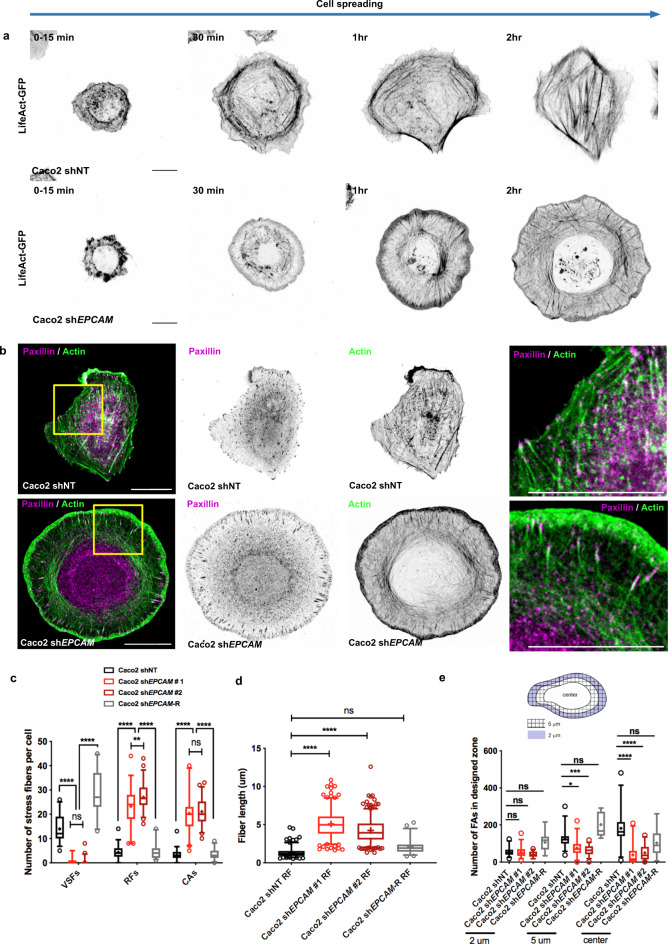
EpCAM participates in the generation of ventral stress fibers in Caco2 cells. **a** Time-lapse images of actin cable rearrangement during cell spreading and polarity acquisition in LifeAct-GFP-transfected control and *EPCAM*-KD cells. Scale bar, 5 μm. **b** Confocal analysis of actin (green) and paxillin (magenta) distributions in control and *EPCAM*-KD cells. Areas boxed in yellow are presented on the right. Projected confocal *z*-stacks are presented. Scale bar, 5 μm. **c** Statistical analysis of the number of ventral stress fibers (VSFs), radial fibers (RFs), and circular arcs (CAs) in control, *EPCAM*-KD cells, and EpCAM rescue. *N* (shNT) = 37 cells, *N* (sh*EPCAM*#1) = 39, *N* = (sh*EPCAM*#2) = 46, (sh*EPCAM*-R) = 27. Whiskers represent the 5–95 percentile confidence intervals. The mean is displayed as a cross. Two-way ANOVA and Tukey’s multiple comparisons test for each stress fiber (SF) type. **p = 0.0053, ****p < 0.0001. **d** Statistical analysis of the length of RFs in control, *EPCAM*-KD cells, and EpCAM rescue. The whiskers of the box plot represent the 5–95 percentile confidence intervals; the mean is displayed as a cross. *N* (shNT) = 36 cells, *N* (sh*EPCAM*#1) = 35, *N* (sh*EPCAM* #2) = 43, *N* (sh*EPCAM*-R) = 27; *n* (shNT) = 160 RFs, *n* (sh*EPCAM*#1) = 223, *n* (sh*EPCAM*#2) = 312, *n* (sh*EPCAM*-R) = 49. Kruskal–Wallis test and Dunn’s multiple comparison test, ****p < 0.0001. **e** Statistical analysis of FA density in the delimited 2 μm, 5 μm from the cell periphery, or center area. Mean number of FAs in the 2 μm area for shNT cells = 55.03 ± 22.96 (mean ± SD), sh*EPCAM*#1 = 54.50 ± 28.82, sh*EPCAM*#2 = 40.93 ± 15.67, shEpCAM-R = 106.5 ± 53.17. In the 5 μm area for shNT cells = 124.4 ± 45.24, sh*EPCAM*#1 = 76.40 ± 42.06, sh*EPCAM*#2 = 63.97 ± 25.80, shEpCAM-R = 201.7 ± 63.43, in the center area for shNT cells = 181.5 ± 95.06, sh*EPCAM*#1 = 38.87 ± 52.95, sh*EPCAM*#2 = 49.73 ± 40.90, shEpCAM-R = 103.0 ± 72.65. *N* (shNT) = 31 cells, *N* (sh*EPCAM*#1) = 30, *N* (sh*EPCAM* #2) = 30, *N* (shEpCAM-R) = 11 s. Whiskers represent the 5–95 percentile confidence intervals. The mean is displayed as a cross. Kruskal–Wallis test and Dunn’s multiple comparison test. *p = 0.0159, ***p = 0.0008, ****p < 0.0001. For each experiment, three independent experiments were carried out. ns non-significant.

Many studies have reported that acquisition of single-cell polarity is achieved through changes in the ordering of actin cytoskeleton and FAs^11,24,70,71^. In line with this idea, monitoring the actin dynamics revealed that the actin cables reorganized as the cells spread and acquired a front–rear axis in control epithelial cells (Fig. 2a and Supplementary Movie 4). First, CAs formed at the boundary between the lamellipodium and lamella. This event was coupled with the appearance of RFs within 30 min; they gave rise to SFs within 2 h, as previously described in fibroblasts^15,72,73^ (Fig. 2a and Supplementary Movie 4). In contrast, *EPCAM*-KD cells generated CAs and RFs but the fiber dynamics was impacted (Supplementary Movie 5). Although the CAs were generated in a similar period of time as in the control cells (~10–20 min), they were longer lived in *EPCAM*-KD cells (lifetime of 16 min +/− 5.92 in control cells versus 33.6 + /− 24.86 in mutant cells) and often still visible 1 h after plating (Supplementary Fig. 2a, b). The mutant cells kept this organization of actin cables in an apparent frozen state during the course of the experiment (Supplementary Movie 5). This result suggested that the process of SF formation could be impaired in *EPCAM*-KD cells. Accordingly, we first analyzed the actin network architecture with the distribution of the paxillin FA marker 2 days after seeding. While the control cells had an important pool of VSFs, *EPCAM*-silenced cells displayed very few (Fig. 2b, c). However, no notable difference in VSFs’ and CAs’ length between control and *EPCAM*-KD cells was observed (Supplementary Fig. 2c, d). *EPCAM*-KD cells instead contained a dense central network of CAs and longer RFs (Fig. 2b–d). The specificity of these abnormalities was tested with rescue experiments by transfecting a shRNA-resistant EpCAM-GFP construct into *EPCAM*-depleted cells. The rescue of EpCAM expression induced a recovery of the number of SF subtypes and RF length (Fig. 2c, d and Supplementary Fig. 2e). We then analyzed the co-distribution of α4-actinin and myosin-IIA. The SFs of the control cells were cross-linked by a periodic distribution of α4-actinin that alternated with myosin-IIA (Supplementary Fig. 2f). In contrast, in *EPCAM*-KD cells, α4-actinin accumulated on RFs devoid of myosin-IIA, which was enriched along CAs (Supplementary Fig. 2f). Their distribution was consistent with the composition of canonical RFs and CAs described in fibroblasts and osteosarcoma cell lines^13,69^. Moreover, since the RFs were predominantly present in *EPCAM*-KD cells, the connected FAs were radially oriented and located mostly in a 5-μm belt at the periphery of these cells (Fig. 2e). Nevertheless, the FA’s length was only slightly increased in the absence of EpCAM (Supplementary Fig. 2g, h). In addition, β1-integrin, zyxin, and the tension-sensitive proteins talin and vinculin were still located to the radially oriented FAs in the absence of EpCAM (Supplementary Fig. 2i). We concluded that the loss of EpCAM had very little impact on the composition and morphology of FAs per se but more on their localization and number. FA number and localization was recovered after EpCAM rescue (Fig. 2e and Supplementary Fig. 2e). In summary, the results showed that *EPCAM* KD disrupts the organization of the actin network and subsequently the location of FAs in single epithelial cells, and suggested that SF formation could be impaired in the absence of EpCAM.

### Loss of EpCAM changes the cell stiffness and traction force application

**Fig. 3:**
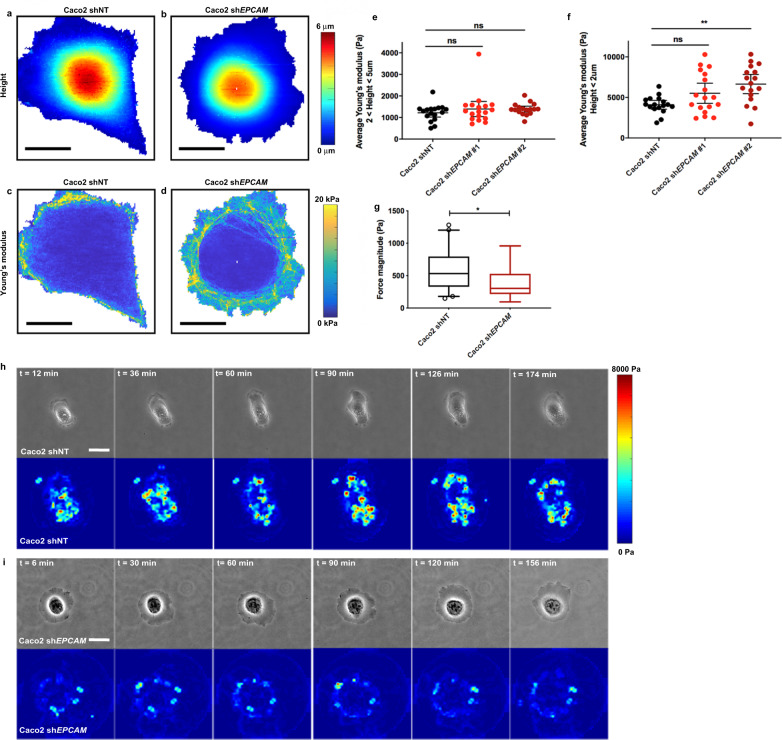
Loss of EpCAM induces cell mechanical changes. **a**–**d** Maps showing the color-coded cell topography (**a**, **b**) and Physics LUT table color-coded local Young’s modulus (**c**, **d**) for a representative control (shNT) (**a**, **c**) and *EPCAM*-KD (sh*EPCAM*) (**b**, **d**) cells. Color scale bar in **a** and **b** indicates the cell height (μm). Color scale bar in **c** and **d** indicates the force magnitude (kPa). Scale bar, 20 μm. **e**, **f** Average Young’s modulus in the region of height in the range of 2–5 μm (**b**) or in the region of height <2 μm (**c**) for each cell. *N* (shNT) = 17 cells, *N* (sh*EPCAM*#1) = 18, and *N* (sh*EPCAM*#2) = 17. Each data point is shown, with the mean and 5–95 percentile confidence intervals. Kruskal–Wallis test with Dunn’s multiple comparison tests, ns = non-significant, **p = 0.0039. **g** Statistical analysis of the mean traction forces measured in control and *EPCAM*-KD cells. *N* (shNT) = 40 cells, *N* (sh*EPCAM*#2) = 13. Whiskers represent the 5–95 percentile confidence intervals. The mean is displayed as a cross. Two-sided Mann–Whitney test, *p = 0.0223. **h**, **i** Representative phase-contrast and Physics LUT table color-coded map images of traction forces (Pa) exerted by control (**h**) and *EPCAM*-KD (**i**) cells. Color bar indicates the force magnitude (Pa). Scale bar, 5 μm. For each experiment, three independent experiments were carried out. ns non-significant.

Given the differences in tension-bearing and force-generating properties between actin fiber subtypes^17^, we reasoned that the modified organization of the actin network could cause an alteration in the mechanical properties of *EPCAM*-KD cells and could explain their defective migration behavior. To do so, atomic force microscopy (AFM) experiments were performed in a rapid force–volume mode to measure the local stiffness across different cell regions. To obtain high spatial resolution, a probe with sharp apex radius (~35 nm) indented the sample at a high velocity (100 μm/s) and force–indentation curves were fit taking into account the finite sample thickness (Supplementary Fig. 3a–c)^74^. However, contributions from nonlinear elastic, anisotropic, and viscoelastic effects are likely amplified using this approach, and the stiffness values measured are an apparent elastic modulus as opposed to the Young’s modulus. In particular, viscoelastic effects due to the high indentation velocity have been shown to result in an apparent stiffening^75^. Here, similar rigidity was detected in the central region of the cell (i.e., cell heights between 2 and 5 μm) in control and *EPCAM*-KD cells (Fig. 3a–e). Nevertheless, as expected, different rigidities were measured in the cell peripheral regions (cell height <2 μm, ~60% increase in apparent elastic modulus for *EPCAM*-KD cells; Fig. 3a–d, f), demonstrating that EpCAM depletion resulted in a higher stiffness of the protrusion. This difference was largely due to the higher density of contractile CAs in unpolarized *EPCAM*-KD cells resolved by the AFM nanoindentation (Fig. 3a–d). These findings suggested that EpCAM may play a role in regulating intracellular stiffness through its action on the actin cytoskeleton, its depletion leading to stiffer and less deformable cells, limiting cell polarization, as previously suggested in a rigidity-sensing mechanism^76^.

In addition, we measured the impact of actin network remodeling in mutant cells on their ability to generate traction forces on the substrate by traction force microscopy (TFM) experiments^77^. The data revealed that *EPCAM*-KD cells exerted lower traction forces on the substrate than control cells (mean of 388.1 and 577.4 Pa, respectively) (Fig. 3g). Furthermore, time-lapse analysis of the TFM data showed that the control cells generated traction forces dynamically, probing the substrate, while the low forces developed by the *EPCAM*-KD cells were maintained throughout the experiment (Fig. 3h, i). Thus, changes in the organization of the actin cytoskeleton between the control and mutant cells impacted the level and distribution of traction forces. Consequently, the absence of contractile SFs at cell–substrate adhesion sites in *EPCAM*-KD leads to the generation of lower forces, as previously observed in similar cases^11^. Altogether, the data demonstrate that deprivation of EpCAM alters the mechanical properties of single epithelial cells.

### EpCAM expression is sufficient to induce VSF formation

**Fig. 4:**
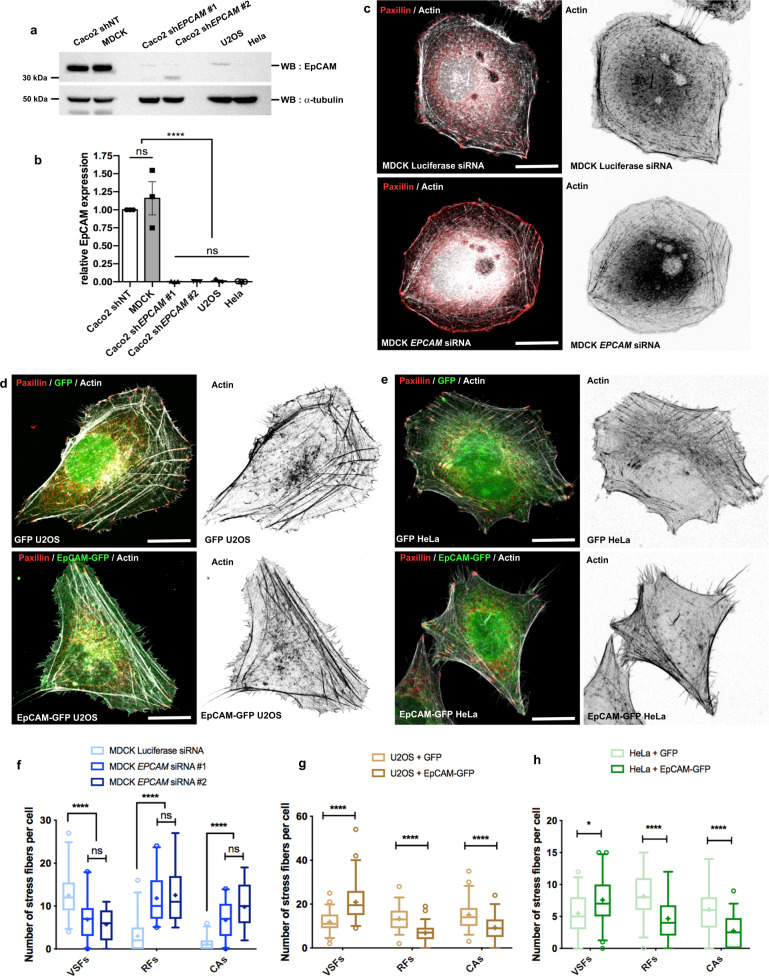
EpCAM expression stimulates the formation of stress fibers in diverse cell types. **a** Western blot analysis of EpCAM expression in control Caco2 (shNT), MDCK, *EPCAM*-KD Caco2 (sh*EPCAM*#1 and #2), U2OS, and HeLa cells. α-Tubulin was used as a loading control. **b** Quantification of EpCAM expression in control Caco2, MDCK, *EPCAM*-KD, U2OS, and HeLa cells. One-way ANOVA test with Tukey’s multiple comparison test, ****p < 0.0001. Data are mean ± SD. **c**–**e** Confocal analysis of actin and paxillin in control and *EPCAM* siRNA-treated MDCK cells (**c**), in GFP- and EpCAM-GFP-transfected U2OS cells (**d**), and in GFP- and EpCAM-GFP-transfected HeLa cells (**e**). Projected confocal *z*-stacks are presented. Scale bar, 5 μm. **f** Statistical analysis of the number of ventral stress fibers (VSFs), radial fibers (RFs), and circular arcs (CAs) in control (Luciferase siRNA) or *EPCAM*-silenced (*EPCAM* siRNA) MDCK cells. *N* (MDCK Luciferase siRNA) = 33 cells, *N* (MDCK *EPCAM* siRNA#1) = 21, *N* (MDCK *EPCAM* siRNA#2) = 11. Whiskers represent the 5–95 percentile confidence intervals. The mean is displayed as a cross. Two-way ANOVA test with Turkey’s multiple comparison test, ****p < 0.0001. **g** Statistical analysis of the number of VSFs, RFs, and CAs in U2OS cells transfected with either GFP (GFP U2OS) or EpCAM-GFP (EpCAM-GFP U2OS). *N* (GFP U2OS) = 50 cells, *N* (EpCAM-GFP U2OS) = 58. Whiskers represent the 5–95 percentile confidence intervals. The mean is displayed as a cross. Two-way ANOVA and Bonferroni’s multiple comparisons test, ****p < 0.0001. **h** Statistical analysis of the number of stress fibers, radial fibers, and circular arcs in HeLa cells transfected with either GFP (GFP HeLa) or EpCAM-GFP (EpCAM-GFP HeLa). *N* (GFP HeLa) = 36 cells, *N* (EpCAM-GFP HeLa) = 44. Whiskers represent the 5–95 percentile confidence intervals. The mean is displayed as a cross. Two-way ANOVA and Bonferroni’s multiple comparisons test, *p = 0.0157, ****p < 0.0001. For each experiment, three independent experiments were carried out. ns non-significant.

EpCAM is expressed in several simple epithelia, and we investigated whether its impact on SF organization could be observed in cell assemblies. A similar mis-arrangement of FAs and actin cables was observed in *EPCAM*-KD cell islands (Supplementary Fig. 3d), demonstrating that the impact of EpCAM on cell–substrate adhesion and actin network architecture also takes place when cell–cell contacts are formed. To determine whether this function of EpCAM could be generalized, we probed the SF organization of other cell types reasoning that those expressing EpCAM should behave like our control clone while the others would mimic our *EPCAM*-KD clones. We used MDCK renal epithelial cells, which have an EpCAM expression level comparable to that of the Caco2 control cells (Fig. 4a, b). We also used U2OS osteosarcoma and the EpCAM-negative HeLa endometrial epithelial cell line, which display an absence of EpCAM similar to Caco2 *EPCAM*-KD cells (Fig. 4a, b)^78,79^. While MDCK control cells showed tangential SFs, their formation was prevented after *EPCAM* small interfering RNA (siRNA)-mediated silencing (Supplementary Fig. 4 and Fig. 4c, f). Conversely, while U2OS and HeLa cells generally displayed a large majority of RFs and CAs, as previously reported^13^, the ectopic expression of EpCAM was sufficient to drive actin fiber rearrangement in these cell lines (Fig. 4d, e, g, h). We concluded that EpCAM participates in a general cell-autonomous regulatory mechanism for SF formation.

### Aberrant contractile activity is at the origin of SF defects and cell polarity failure

**Fig. 5:**
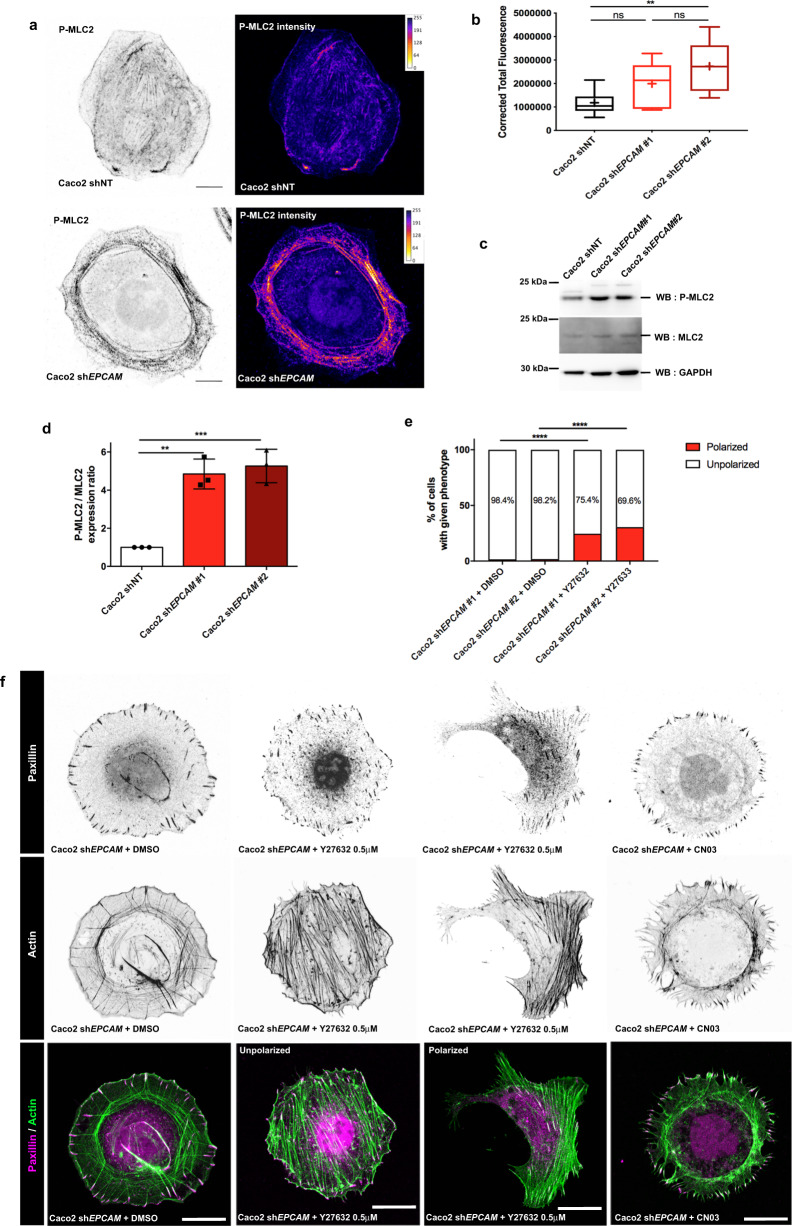
Defective cell contractility activity and distribution is responsible of the development of the *EPCAM*-KD phenotype. **a** Confocal analysis of the distribution of P-MLC2 in control and *EPCAM*-KD Caco2 cells. Projected confocal *z*-stacks are presented. P-MLC2 signal intensity is color-coded with Fire LUT table from ImageJ on the right panel; color scale bar indicates the gray value intensity. Scale bar, 5 μm. **b** Statistical analysis of the corrected total fluorescence for P-MLC2 in control and *EPCAM*-KD cells. *N* (shNT) = 10 cells, *N* (sh*EPCAM*#1) = 10, *N* (sh*EPCAM*#2) = 10. The whiskers represent the 5–95 percentile confidence intervals, and the mean is shown as a cross. One-way ANOVA test with Tukey’s multiple comparison test, **p = 0.0012. **c** Western blot analysis of MLC and P-MLC amounts in control and *EPCAM*-KD cells. GAPDH was used as a loading control. **d** Statistical analysis of P-MLC2 amount relative to MLC2 in control and *EPCAM*-KD cells. One-way ANOVA test, **p = 0.0005, and Tukey’s multiple comparison tests. **p = 0.0011, ***p = 0.0006. Data are mean ± SD. **e** Statistical analysis of the number of unpolarized- and polarized-shaped sh*EPCAM*#1 and #2 cells after DMSO or Y-27632 0.5 μM treatment. The exact number (and %) of cells in each category is available in Supplementary Table 4. *N* (DMSO sh*EPCAM*#1) = 192 cells, *N* (DMSO sh*EPCAM*#2) = 111, *N* (Y-27632 sh*EPCAM*#1) = 199, *N* (Y-27632 sh*EPCAM*#2) = 207. Chi-square test, ****p < 0.0001. **f** Confocal analysis of paxillin (magenta) and actin (green) in *EPCAM*-KD cells upon DMSO, blebbistatin 2 μM, Y-27632 0.5 μM, CN03 1 μg/ml, or SMIFH2 2 μM treatment for 1 h. Projected confocal *z*-stacks are presented. Scale bar, 5 μm. For each experiment, three independent experiments were carried out. ns non-significant.

Several mechanisms were put forward to explain SF maturation. Among them, α4-actinin dynamics are known to influence the incorporation of myosin-IIA into SFs during their maturation. α4-Actinin was previously reported as a binding partner of EpCAM^48^. Since Myosin-IIA and α4-actinin bindings on the SF are mutually exclusive, we hypothesized that *EPCAM*-KD could prevent the recruitment of myosin-IIA. However, in our hands, no interaction between EpCAM and α4-actinin was detected after co-immunoprecipitation (Supplementary Fig. 5a). Moreover, *ACTN4*-KD by siRNA could not restore the presence of VSFs in *EPCAM*-KD cells (Supplementary Fig. 5b, c). Previous studies have described that the intensity and distribution of cell contractility are modulated by EpCAM in epithelial assemblies^53,50^. Therefore, we focused on contractility mechanisms to determine whether they are involved in the development of the *EPCAM*-KD phenotype in single cells. As shown in Supplementary Fig. 2f, myosin-IIA remains associated with CAs in mutant cells. To evaluate the contractile ability of myosin-IIA, we performed an immunostaining directed against the phosphorylated form of the myosin regulatory light chain (P-MLC2) (Fig. 5a). In absence of EpCAM, the P-MLC2 signal was intensified (Fig. 5a, b), as previously described in Caco2 cell clusters (Magzhal et al.^50^). In addition, the P-MLC2 signal was concentrated along CAs in *EPCAM*-KD cells compared to control cells (Fig. 5a, b). The increase in P-MLC2 was confirmed by western blot (WB), where P-MLC2 levels increased relative to the total amount of MLC2 in *EPCAM*-KD cells (Fig. 5c, d). These data demonstrated that actomyosin activity is elevated but restricted to CAs, creating a uniform hypercontractile ring at the cell cortex in single *EPCAM*-silenced cells.

To test whether the local hyperactivity of the actomyosin network was at the origin of SF and FA abnormalities and to identify the signaling pathway involved, we subjected the *EPCAM*-KD cells to various drug treatments affecting cell contractility and actin polymerization. Reducing the ATPase activity of myosin-II by a conventional treatment of 10 μM blebbistatin resulted in a total disappearance of RFs and CAs, as previously described (Supplementary Fig. 6a)^15,80^. However, treatment with 2 μM blebbistatin caused a decrease in the number of CAs and the swirling of actin cables with the development of a few SFs, as well as more numerous and centrally localized FAs (Supplementary Fig. 6a). Similarly, a treatment with 10 μM Y27632 to reduce ROCK1 activity abolished the formation of RFs and CAs (Supplementary Fig. 6a). But a low dose of Y-27632 (0.5 μM) caused the disappearance of RFs, the formation of linear SFs, and the redistribution of FAs in the cell (Fig. 5f). In addition, almost one-third of *EPCAM*-KD cells treated with a low dose of Y-27632 returned to a polarized shape (Fig. 5e, f). We concluded that mild adjustments in the level of CA contractility is capable of triggering partial or total recovery of the SF maturation and cell polarization. Interestingly, these data pointed to RhoA signaling. To estimate the degree of RhoA involvement in the development of the *EPCAM*-KD phenotype, we evaluated the level of endogenous active RhoA with fluorescence resonance energy transfer (FRET) experiments in control or *EPCAM*-silenced cells. We used the FRET probe developed by Matsuda and colleagues^81^, which consists of yellow fluorescent protein (YFP), Rhotekin Rho-binding domain (RBD), and cyan fluorescent protein (CFP). Binding of endogenous GTP-loaded RhoA to the RBD provokes the separation of the YFP and CFP tags, resulting in a decrease of the FRET ratio (Supplementary Fig. 6b). Here, *EPCAM* silencing caused a significant decrease in the FRET ratio (Supplementary Fig. 6c, d). Moreover, the overactivation of RhoA by treatment of mutant cells with the RhoA activator CN03 amplified the *EPCAM*-KD phenotype (Fig. 5f). These results reflected an increase in RhoA activity in the absence of EpCAM.

Furthermore, as shown in Fig. 2d, *EPCAM*-KD cells display long RFs. This data prompted us to test the activity of formins, as they are involved in RF polymerization and are also RhoA effectors^82^. Along this line, we evaluated their activity in *EPCAM*-depleted cells using the pan-formin inhibitor SMIFH2, which prevents formin nucleation as well as their affinity for the actin barbed ends where the filament elongation occurs^83^. Treatment with low-doses of SMIFH2 reduced RF size and the depth of cell protrusion, but failed to restore SF development and cell shape remodeling (Supplementary Fig. 6e). Taking into account the crosstalk between small GTPases and the presence of the large lamellipodia of *EPCAM*-KD cells^84,85^, we also investigated whether an imbalance in Rac1 activity could participate in the *EPCAM*-KD phenotype. Although treatment with the Rac1 inhibitor NSC23766 reduced the size of the lamellipodium, it did not rescue a correct arrangement of the actin cables and adhesive structures (Supplementary Fig. 6e). Additionally, inhibition of Arp2/3, an effector of Rac1, with a CK666 treatment had no effect on *EPCAM*-KD cells^86^. Finally, we also tested the possible involvement of the myosin light chain kinase (MLCK) pathway, a parallel pathway for myosin-II phosphorylation^87^. Treatment with ML-7, which inhibits MLCK activity, had no obvious impact on *EPCAM*-silenced cells (Supplementary Fig. 6e). Together, the results indicated that a major participation of RhoA signaling occurs toward the regulation of cell contractility rather than actin polymerization in the EpCAM-dependent mechanism. We concluded that local actomyosin hyperactivity is at the origin of the defects in SF maturation and polarity acquisition induced by the *EPCAM* KD. Our results show that EpCAM could act upstream of the actomyosin activity, probably at the level of RhoA signaling.

### Cortical zone of active RhoA is remodeled during epithelial cell spreading and requires EpCAM

To determine the impact of EpCAM on Rho signaling, we transfected Caco2 cells with wild-type (wt) RhoA (GFP-Rho), a constitutively active mutant (GFP-RhoG14V), or a dominant-negative mutant of RhoA (GFP-RhoT19N). In control cells, the expression of mutant forms of Rho destabilized the actin network and FA organization, as expected (Supplementary Fig. 7). The expression of GFP-RhoG14V led to an increase in FA size, similar to the phenotype of *EPCAM*-KD FAs, but did not affect their subcellular localization. The GFP-RhoG14V-treated cells also enhanced VSF formation, unlike *EPCAM*-KD cells. Moreover, the expression of GFP-RhoT19N caused a reduction in the number and concentration of FAs at the cell periphery, as in *EPCAM*-KD cells; however, it did not provoke any obvious change in FA size. Additionally, the GFP-RhoT19N mutant induced a decrease in the overall SF content, but VSFs were still present at the cell periphery and we could not detect any clear effect on the arrangement of RFs or CAs (Supplementary Fig. 7). Thus, the expression of the two mutant forms of Rho in control cells only partially reproduced some aspects of the *EPCAM*-KD phenotype.

Furthermore, the introduction of GFP-RhoG14V into *EPCAM*-silenced cells worsened their KD phenotype, which then resembled that of CN03-treated cells (Fig. 5f). After transfection with GFP-RhoT19N, no obvious changes were observed in the arrangement of actin and FAs in *EPCAM*-KD cells (Supplementary Fig. 7). Together, these data led us to conclude that RhoA activity could contribute to the emergence of defects after *EPCAM* silencing. However, RhoA modulation using drugs or constitutively active or inactive mutants did not seem to be sufficient to explain the *EPCAM*-KD phenotype, and we thought that a spatial and/or a temporal factor might be missing in this analysis.

**Fig. 6:**
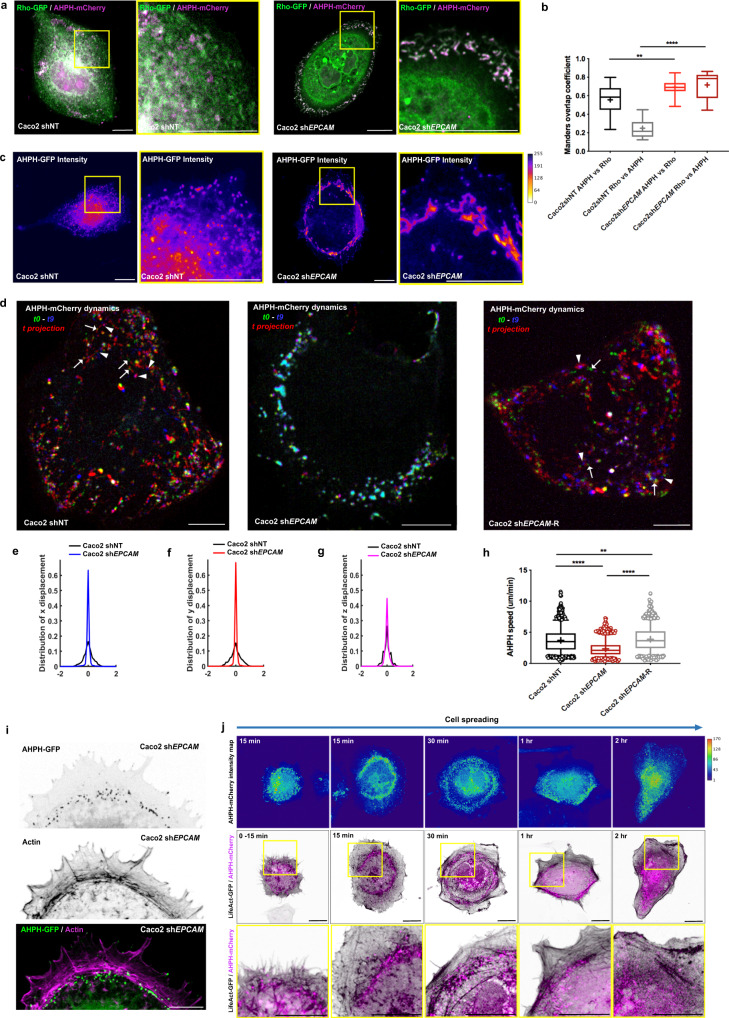
*EPCAM* depletion leads to compromised dynamics of GTP-RhoA-positive vesicles. **a** Confocal analysis of the distribution of RhoA-GFP (green) together with AHPH-mCherry (magenta) in control and *EPCAM*-KD cells. Areas boxed in yellow are presented on the right. Projected confocal *z*-stacks are presented. Scale bar, 5 μm. **b** Quantification of the proportion of the AHPH-mCherry probe overlapping with total RhoA. Manders overlap coefficient for AHPH-mCherry versus RhoA-GFP in shNT cells = 0.5558 ± 0.04 and in sh*EPCAM* cells = 0.6913 ± 0.02. Whiskers represent the 5–95 percentile confidence intervals. The mean is displayed as a cross. Manders overlap coefficient for RhoA-GFP versus AHPH-mCherry in shNT cells = 0.2485 ± 0.03, and in sh*EPCAM* cells = 0.7165 ± 0.03. *N* (shNT) = 5 cells, *N* (sh*EPCAM* #1) = 5. Two-sided Mann Whitney test, **p = 0.0077; ****p < 0.0001. Values are mean ± s.e.m. **c** AHPH-GFP intensity maps were generated in control and *EPCAM*-KD cells with Fire LUT table from ImageJ; color scale bar indicates the gray value intensity. Areas boxed in yellow are presented on the right. Projected confocal *z*-stacks are presented. Scale bar, 5 μm. **d** Color-coded *t*-projection of 10 frame time-lapse series of AHPH-mCherry in control, *EPCAM*-KD, or EpCAM-rescued (shEpCAM-R) cells. The first image (t0) is false-colored green, the last image (t9) is false-colored in blue, and the intervening time points (t2-8) are submitted to *t*-projection and shown in red (*t*-projection). Areas boxed in yellow are presented on the right. Arrows point at the position of some AHPH compartment at the beginning of the time-lapse series, whereas the arrowheads point to the position of the corresponding AHPH compartment at the end of the time-lapse series. Scale bars, 10 μm. **e**–**g** Analysis of the displacement of the AHPH-mCherry compartments in the *x* (**e**), *y* (**f**), and *z* (**g**) direction in control or *EPCAM*-KD cells. **h** Statistical analysis of the speed of AHPH-mCherry compartments in control, *EPCAM*-KD, or EpCAM-rescued cells. The whiskers of the box plot represent the 5–95 percentile confidence intervals; the mean is displayed as a cross. *N* (shNT) = 1827 AHPH-positive vesicles, *N* (sh*EPCAM*) = 1234, *N* (sh*EPCAM*-R) = 1689. Kruskal–Wallis test (*p* < 0.0001) and Dunn’s multiple comparison tests, **p = 0.0037, ****p value < 0.0001. **i** Confocal analysis of the distr**i**bution of AHPH-GFP (green) and actin cables (magenta) in the protrusion of *EPCAM*-KD cells. Projected confocal *z*-stacks are presented. Scale bar, 5 μm. **j** Confocal analysis of the distribution of AHPH-mCherry (magenta) and actin (black) in control cells during cell polarization and maturation of stress fibers from 0 to 2 h. Areas boxed in yellow are presented on the bottom panel. In the upper panel, AHPH-mCherry intensity maps have been color-coded using the Physics LUT from ImageJ; color scale bar indicates the gray value intensity. Projected confocal *z*-stacks are presented. Scale bar, 5 μm. For each experiment, three independent experiments were carried out. ns non-significant.

We thus assessed the subcellular localization of the GTP-loaded form of RhoA by taking advantage of the fluorescent location biosensor AHPH, which is derived from the C-terminus end of anillin^88,89^. mCherry-tagged AHPH partially overlapped with total Rho-GFP (Supplementary Fig. 8a) and co-distributed significantly with other RhoA location biosensors derived from the effectors ROCK1 and mDia (ROCK1-GBD-GFP and mDia-GBD-GFP, based on the GTP-RhoA-binding domain (GBD) of the respective protein) (Supplementary Fig. 8b, c)^90^. Moreover, the expression of a form of AHPH mutated in its RBD domain and therefore unable to bind GTP-RhoA (AHPH^A740D^-GFP) generated a intracellular pattern different from the wt form of AHPH (Supplementary Fig. 8d)^89,91^. These data demonstrated the specificity of the tagged form of AHPH for GTP-RhoA signal and confirmed that it could be used to probe the dynamics of active RhoA. First of all, we carefully examined the spatial distribution of total RhoA and its GTP-loaded form using Rho-GFP and AHPH-mCherry, respectively (Fig. 6a). The control cells showed partial colocalization of RhoA-GFP and AHPH-mCherry in intracellular structures (Fig. 6a, b), as previously described in endothelial and neuronal cells^91–93^. On the contrary, *EPCAM*-KD cells displayed an accumulation of RhoA and its active form in large tubular compartments in the lamella (Fig. 6a–c) and their colocalization increased up to 70% (Fig. 6b). These results suggested that a slowing down in the GTPase cycle could take place, maintaining RhoA in its GTP-loaded form in the absence of EpCAM. Defects in RhoA dynamics would support this hypothesis, we therefore followed the reporter’s behavior in live. The broad GTP-RhoA displacement profiles revealed complex intracellular dynamics in the protrusion of control cells (Fig. 6d and Supplementary Movie 6). However, in the absence of EpCAM, the movement of the active RhoA probe was altered with a decrease in speed and reduced displacement (Fig. 6d–h and Supplementary Movie 7). Transfection of a shRNA-resistant EpCAMr-GFP construct into KD cells restored a correct distribution and dynamics for AHPH (Supplementary Fig. 9a, Fig. 6d, g, and Supplementary Movie 8). These data showed that EpCAM is required for the dynamics of GTP-RhoA in the cell protrusion.

Additionally, the AHPH-positive compartments were enriched in the vicinity of the CAs in *EPCAM*-KD cells (Fig. 6i). This observation led us to consider a positive correlation between GTP-RhoA dynamics and actin cable remodeling in Caco2 cells. Although the typical transitions in cytoskeletal rearrangement during cell spreading and polarity acquisition are well described in literature, an overall spatial and temporal study of the contractility signaling in this context is still missing. By analyzing the spatio-temporal dynamics of AHPH-mCherry, we determined that active RhoA exhibited significant changes in distribution during cell shape remodeling (Fig. 6j). For the first 15 min after seeding, while no actin cable could be distinguished yet, GTP-RhoA-positive compartments were located around the nucleus in the round-shaped cells. While the spreading intensified with the clear expansion of the protrusions in circular-shaped cells, GTP-RhoA became concentrated in a central ring and co-distributed with a dense meshwork of short actin filaments. At 30 min after seeding, the CAs formed near the central ring of active RhoA. In the final stage, starting from 1 h after seeding, the central ring aspect disappeared and AHPH was instead scattered homogeneously in the cytoplasm. The remodeling of GTP-RhoA distribution was concomitant with the acquisition of SFs and a polarized cell shape. Thus, we concluded that indeed a correlation exists between GTP-RhoA dynamics and actomyosin rearrangement. The reorganization of the cortical zone of active RhoA allows the remodeling of actin fibers and the polarized cell reshaping, pivotal steps which are blocked in the absence of EpCAM.

### EpCAM regulates the progression of active RhoA in endosomal recycling platforms

**Fig. 7:**
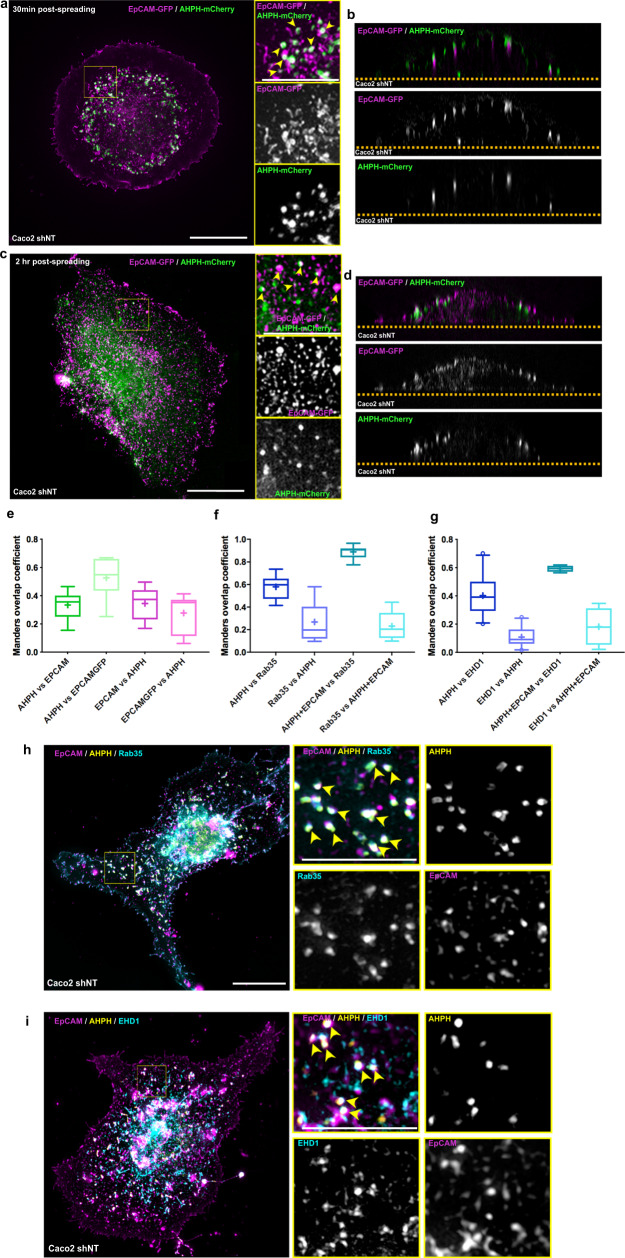
EpCAM and active RhoA co-evolve in Rab35/EHD1-positive endosomal compartments. **a**–**d** 3D-SIM microscopy analysis of EpCAM-GFP (magenta) and AHPH-mCherry (green) in unpolarized single cells during cell spreading (**a**, **b**) and polarity acquisition (**c**, **d**) in the *xy* plane (**a**, **c**) or the *xz* plane (**b**, **d**). Areas boxed in yellow are presented on the right, where arrowheads point to colocalizations. Collagen substrate is delimited by an orange dotted line. Arrows point on colocalizations. Scale bar, 5 μm; inset scale bar, 2.5 μm. **e** Quantification of the Manders overlap coefficient between AHPH-mCherry versus endogenous EpCAM, AHPH-mCherry versus EpCAM-GFP, endogenous EpCAM versus AHPH-mCherry, and EpCAM-GFP versus AHPH-mCherry in polarized single cells based on confocal microscopy acquisitions. Whiskers represent the 5–95 percentile confidence intervals. The mean is displayed as a cross. **f** Quantification of the Manders overlap coefficient between AHPH-GFP versus Rab35-RFP, Rab35-RFP versus AHPH-GFP, AHPH-GFP+endogenousEpCAM versus Rab35-RFP, and Rab35-RFP versus AHPH-GFP+endogenousEpCAM in polarized single cells based on confocal microscopy acquisitions. Whiskers represent the 5–95 percentile confidence intervals. The mean is displayed as a cross. **g** Quantification of the Manders overlap coefficient between AHPH-mCherry versus EHD1-GFP, EHD1-GFP versus AHPH-mCherry, AHPH-mCherry+endogenousEpCAM versus EHD1-GFP, and EHD1-GFP versus AHPH-mCherry+endogenousEpCAM in polarized single cells based on confocal microscopy acquisitions. Whiskers represent the 5–95 percentile confidence intervals. The mean is displayed as a cross. **h**, **i** 3D-SIM microscopy analysis of EpCAM (magenta), AHPH-GFP (yellow), and Rab35-RFP or EHD1-GFP (blue) in polarized single cells. Areas boxed in yellow are presented on the right, where arrowheads point to colocalizations. Scale bar, 5 μm; inset scale bar, 2.5 μm. For each experiment, three independent experiments were carried out. ns non-significant.

Collectively, the data raised a basic question: how does EpCAM ensure proper cell spreading and actin fiber organization? In light of the results described above, we wondered whether EpCAM could act directly on the active RhoA zone remodeling during spreading. By analyzing the distribution of EpCAM with the GTP-RhoA location biosensor, we found that a subpopulation of endogenous EpCAM co-distributed with the RhoA reporter in intracellular compartments. Similar results are seen with an EpCAM-GFP construct (Fig. 7a–e and Supplementary Fig. 9a). This colocalization took place in nonpolarized cells and continued when the cells acquired front–rear polarity (Fig. 7a, b, c, d, respectively), reflecting a close interplay between EpCAM and GTP-RhoA during cell spreading.

**Fig. 8:**
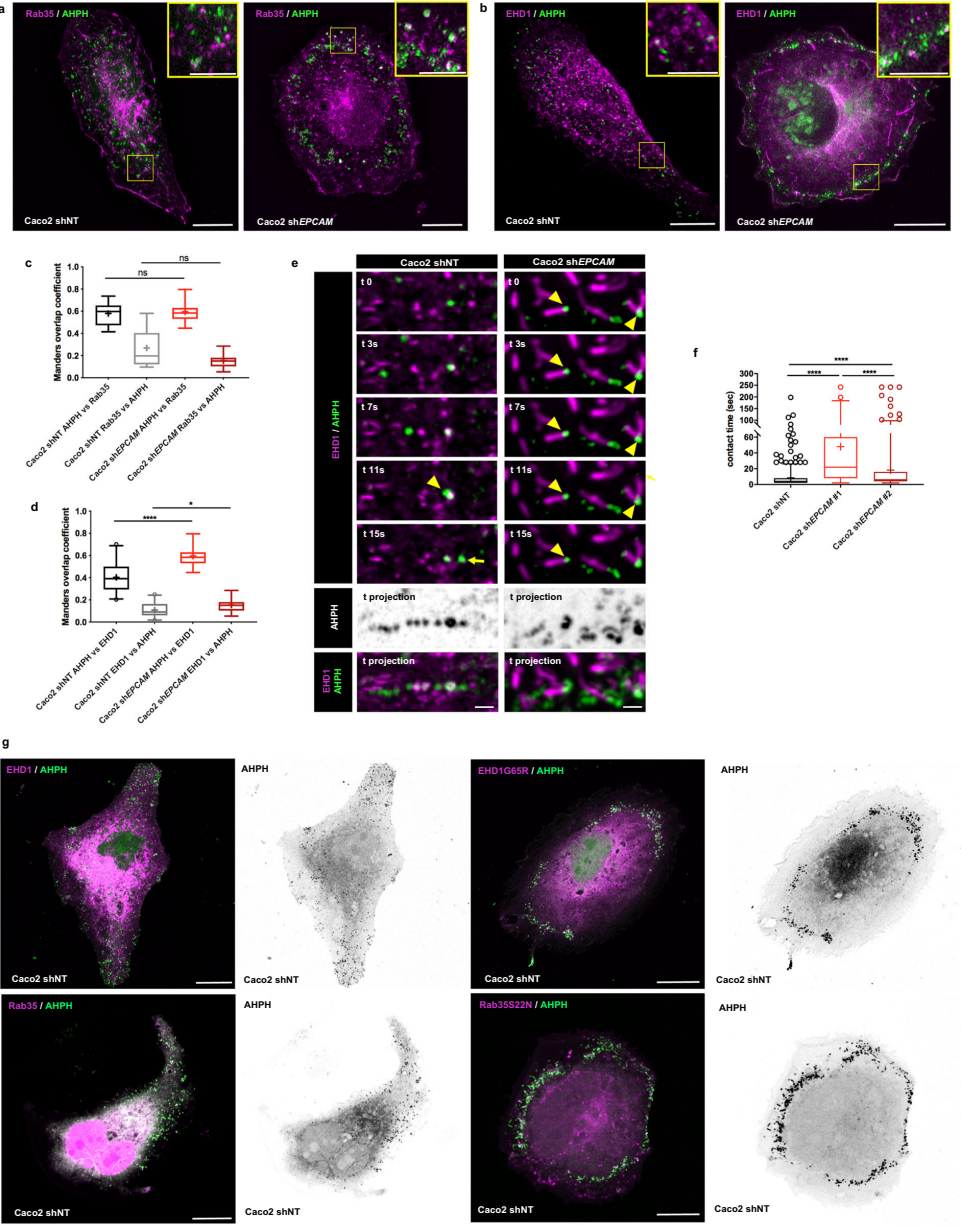
Active RhoA is blocked in Rab35/EHD1-positive endosomal compartments in the absence of EpCAM. **a**, **b** 3D-SIM microscopy analysis of the distribution of Rab35-RFP (**a**, magenta) or EHD1-GFP (**b**, magenta) together with AHPH-mCherry (green) in control and *EPCAM*-KD cells. Areas boxed in yellow are presented in the right corner. Scale bars, 5 μm; inset scale bar, 2 μm. **c** Quantification of the Manders overlap coefficient between AHPH-GFP versus Rab35-RFP or Rab35-RFP versus AHPH-GFP in control or *EPCAM*-KD cells based on confocal microscopy acquisitions. *N* (shNT) = 13 cells, *N* (sh*EPCAM*) = 15. Whiskers represent the 5–95 percentile confidence intervals. The mean is displayed as a cross. Two-sided Mann–Whitney test; for AHPH versus Rab35, *p* = 0.9639; for Rab35 versus AHPH, *p* = 0,1077. **d** Quantification of the Manders overlap coefficient between AHPH-mCherry versus EHD1-GFP or EHD1-GFP versus AHPH-mCherry in control or *EPCAM*-KD cells based on confocal microscopy acquisitions. Whiskers represent the 5-95 percentile confidence interval. The mean is displayed as a cross. *N* (shNT) = 22 cells, *N* (sh*EPCAM*) = 15. Two-sided Mann Whitney test; *p = 0.0379, ****p < 0.0001. **e** Time-laps**e** series and maximum projection (standard deviation) of EHD1-GFP together with AHPH-mCherry in control and *EPCAM*-KD cells. Yellow arrowheads point at the position of colocalized AHPH and EHD1 compartments. Yellow arrow points at an AHPH-positive vesicle at the exit of an EHD1-positive compartment. Scale bars, 200 nm. **f** Statistical analysis of the residence time between the AHPH probe and the EHD1-positive compartments per track in control and *EPCAM*-KD cells. Measurements were made on time-lapse series of 1-s intervals. *N* (shNT) = 8 cells (491 contacts), *N* (sh*EPCAM*#1) = 3 cells (55 contacts), *N* (sh*EPCAM* #2) = 7 cells (213 contacts). Whiskers represent the 5–95 percentile confidence intervals. The mean is displayed as a cross. Kruskal–Wallis test and Dunn’s multiple comparison tests, ****p < 0.0001. **g** Confocal analysis of AHPH-mCherry (green) after EHD1-GFP and EHD1G65R-GFP expression (magenta) or after Rab35-GFP and Rab35S22N-RFP expression (magenta) in control cells. Scale bars, 5 μm. For each experiment, three independent experiments were carried out. ns non-significant.

To determine where this cooperation could take place and to further understand the dynamics of active RhoA, we tested candidate compartments for GTP-RhoA, using fluorescently tagged Rab GTPases as proxies for organelle identity. For instance, GTP-RhoA was barely detected in Rab5-, EEA1-, Rab4-, Rab8-, Rab7-, LAMP1-, or caveolin-1-positive organelles (Supplementary Fig. 9b–i). In addition, Rab11, the canonical marker of endosomal recycling, showed 15% colocalization with AHPH-positive compartments in Caco2 cells (Supplementary Fig. 9b, j); this result contrasted with the high colocalization rates observed for Rab11 and AHPH in neural crest cells^91^. But interestingly, we identified a preferential distribution of GTP-RhoA in an endosomal subfraction specifically controlled by Rab35 and C-terminal Eps15 homology domain-1 (EHD1), with 60 and 40% co-location rates, respectively (Supplementary Fig. 9b, k, l). Rab35 and EHD1 work in fast endocytic recycling at the level of cortical endosomes. Although they are both involved in the same trafficking pathway, they do not interact directly. Rab35 acts during early stages of cargo recycling and promotes the recruitment of its effector MICAL1, which in turn recruits EHD1 on the recycling endosomes. There, EHD1 ensures the scission of tubular endosomes to trigger the recycling of cargoes^94–99^. Thus, in addition to a functional distinction, spatial compartmentalization occurs between Rab35 and EHD1 in the recycling endosomes. In PC12 cells, Kobayashi and Fukuda have shown that the two proteins did not exhibit a total overlap but instead co-located at 80%^97^. Here, vesicle-shaped endosomal compartments contained both Rab35 and EHD1, but the tubular structures appeared to be devoid of Rab35 and instead contained mostly EHD1 (Supplementary Fig. 10a). Moreover, quantification of the colocalization between EHD1 and Rab35 revealed a 40–50% overlap in Caco2 cells (Supplementary Fig. 10b). In consequence, a given percentage of colocalization may not reflect the total amount of AHPH contained in fast recycling endosomes, and we could only conclude that at least 60% of AHPH was located in fast recycling endosomes. Furthermore, the cooperation between RhoA-GTP and EpCAM occurred preferentially in this recycling endosomal pathway, with colocalization rates of 90% in Rab35-positive compartments and 60% in EHD1-positive ones (Fig. 7f, h, g, i, respectively, and Supplementary Fig. 9b). These results showed that GTP-RhoA and EpCAM route together in the cellular cortex and led us to suggest that EpCAM may dictate the progression of RhoA-GTP in the endosomal recycling domains. Following this idea, we evaluated the impact of EpCAM silencing on the progression of GTP-RhoA through the Rab35-/EHD1-positive compartments. A non-significant change was found for the colocalization of AHPH and Rab35 between control and *EPCAM*-KD cells (Fig. 8a, c). However, the proportion of AHPH colocalized in EHD1-positive compartments increased significantly after *EPCAM*-KD (from 40% in control cells up to 60% in mutant cells; Fig. 8b, d), suggesting that GTP-RhoA may remain accumulated there in the absence of EpCAM.

To test the accumulation of GTP-RhoA in tubular recycling endosomes, we further examined the dynamics of AHPH and EHD1 (Supplementary Movie 9). Tracking revealed a short residence time of AHPH in the EHD1-positive compartments in control cells (yellow arrowheads in Fig. 8e, f and Supplementary Movies 9 and 10). It is worth mentioning that AHPH vesicles entering EHD1-positive compartments could be seen leaving them (yellow arrow in Fig. 8e and Supplementary Movie 10). This data suggested that RhoA could remain active during this process and that its inactivation per se might not occur in these endosomal domains. Moreover, a long residence time was observed in *EPCAM*-KD cells, where AHPH was sequestered in the EHD1-positive compartments (yellow arrowheads in Fig. 8e, f and Supplementary Movies 11 and 12). The cortical accumulation of the AHPH probe that we observed in the absence of EpCAM therefore reflected a retention of GTP-RhoA in endosomal compartments during cell spreading. To provide further evidence that this endosomal pathway was required for GTP-RhoA dynamics and cell organization, we used dominant-negative mutant forms of either EHD1 (mutation of glycine 65 to arginine in the P-loop domain of EHD1 that renders EHD1 cytosolic, i.e., EHD1G65R-GFP)^100^ or Rab35 (Rab35S22N-GFP)^95^. Overexpression of the mutant forms in control cells caused the accumulation of AHPH in a circular manner in the cell protrusion, thereby mimicking the effect of *EPCAM*-KD on GTP-RhoA distribution (Fig. 8g). Interestingly, concomitant perturbation of cell shape was observed after expression of EHD1G65R-GFP or Rab35S22N-GFP where mutant cells acquired an isotropic form, as *EPCAM*-KD cells did (Fig. 8g). However, the phenotype of endosomal mutant cells was more severe than that of *EPCAM*-KD cells in terms of actin and FA organization (Supplementary Fig. 10c). In contrast to the defects developed by *EPCAM*-KD cells, the actin network was here barely detectable in the cell body; only a few cables were observed at the cell edge after endosomal mutant expression; in addition, few small FAs developed at the very cell periphery (Supplementary Fig. 10c). This result indicated that the loss of EpCAM would not block Rab35-EHD1 endosomal pathway process per se but rather the progression of GTP-RhoA at this level. In conclusion, our results show that active RhoA is processed on the Rab35/EHD1 cortical endosomal pathway using EpCAM. In addition, the endosomal GTP-RhoA turnover is required to ensure actin cable rearrangement and cell shape changes.

## Discussion

Our study advances the understanding of contractility control during cell reshaping and reveals the involvement of EpCAM in this process. The work that has emerged from our laboratory and others has previously shown that EpCAM behaves as a key player for the spatial organization of the actomyosin network in epithelial tissues and ultimately in the apico-basal polarity and integrity of the monolayer^50,53,62^. EpCAM was originally described as a cell adhesion molecule acting at cell–cell contacts^47,48^. Here, we show that EpCAM is needed for single epithelial cells to structure their actomyosin network and self-organize in a front–rear axis. We therefore posit that the impact of EpCAM is a general, cell-autonomous regulatory mechanism governing epithelial plasticity. We provide further evidence of a direct implication of EpCAM in the regulation of cell contractility. In fact, EpCAM allows the release of GTP-RhoA from cortical endosomal domains.

From a mechanical point of view, what advantage would the expression of EpCAM bring to an epithelial cell? It is interesting to note that fibroblasts or mesenchymal cells, for instance, do not express EpCAM but self-organize spontaneously and show active motion. However, under physiological conditions, EpCAM is expressed in epithelial layers, which, due to their intrinsic nature of interfaces, are under continuous mechanical stimulation and are subject to important remodeling events. Furthermore, EpCAM expression is often increased in tumors of epithelial origin, which are challenging mechanical environments^101–103^. In fact, EpCAM is widely used as a marker for the detection or the isolation of circulating tumor cells derived from ovarian, breast, or colorectal cancers^104,105^. Here, we reveal that EpCAM promotes RhoA dynamics for SF maturation and migration of single epithelial cells. The expression of EpCAM in tumor cells could maximize the robustness of the RhoA cycle during its transit through the fast recycling endosomal pathway, thus ensuring that tensile forces are correctly generated at the cell scale and therefore supporting tumor propagation.

It is well established that RhoA signaling dictates myosin-II-dependent contractility and SF generation and thus controls cell morphogenesis and behavior^106,107^. The coordinated activities of RhoA, Rac1, and Cdc42 take place in the cell leading edge to sustain protrusive activity as well as rear retraction^108^. The development and use of FRET probes showed that RhoA is highly activated in a 2-μm wide band in the leading edge of migrating cells and participates in its protrusive activity, while Rac1 and Cdc42 stabilize the protrusion for directed motion of fibroblasts^85^. Although these studies have been essential to our overall understanding of RhoGTPase functions, FRET analyses provide only a fixed image and low spatial resolution of their activity at a given time (Supplementary Fig. 6b–d). Here, using a fluorescent location biosensor that allows direct tracking of GTP-RhoA^88,89^, our analyses reveal the transient formation of a cortical ring of active RhoA during the early stages of cell spreading. This active RhoA zone is remodeled during late stages of spreading, before the reorganization of the actomyosin network (Fig. 6j). In agreement with several studies that report the importance of a correct balance of contractile forces for spreading and polarity in fibroblasts cells^24,76^, our study unveils a spatiotemporal modulation of RhoA activity during the development of the front–rear axis in epithelial cells. In addition, blocking of the endosomal GTP-RhoA trafficking in EHD1-/Rab35-mutated or *EPCAM*-KD cells impairs the local regulation of RhoA signaling and thus the completion of late cell spreading steps (Figs. 6 and 8, respectively). In conclusion, we propose that a strict coupling occurs between the active RhoA pool and the actomyosin cables remodeling that is necessary for the initiation of front–rear polarity in epithelial cells.

But what drives this timing? In other words, how is this process controlled and what signals trigger the exit of GTP-RhoA from the cortical endosomes during cell spreading? One explanation could be that RhoA trafficking is controlled by mechanical feedback. Previous work from Sheetz and colleagues has shown a sequential mechanical model of cell spreading, where each phase represents a distinct mechanical state of the cells^109^. The early cell spreading phase, called P1, is characterized by continuous protrusive edge activity with the generation of very low traction forces. The P2 phase is described as a slow spreading phase during which FAs form and high membrane tension occurs. Besides, the control of membrane tension seems to be critical for the progression through this P2 phase^110^ and would be ensured by a coordinated regulation of membrane trafficking events and cell contractility^111–113^. For instance, inhibition of myosin-IIA activity keeps fibroblasts locked in P2 phase^69^. Moreover, reduction of the membrane tension triggers the endocytic process^114^. Here, we found that *EPCAM*-KD cells fail to complete P2 phase and display isotropic organization and, to some extent, C-shapes (Fig. 1d-i; Supplementary Movie 3), as do mutant fibroblasts for myosin-IIA^69^ and reminiscent of fish keratocyte migration mode^115^. Keratocytes migrate after a symmetry-breaking event that depends on the balance between cell–substrate adhesive forces and myosin-II activity levels. Migration remains possible under conditions of low contractility if adhesion to the substrate is also reduced^115^. Furthermore, in fibroblasts, loss of myosin-II activity caused a decrease in traction forces while actin polymerization continued, generating large cell protrusions. However, the rate of actin polymerization may decrease in some area of the lamellipodia. Thus, under the effect of membrane tension forces, they collapsed and provoked a symmetry-breaking event in early P2 phase^69^, as we observed prior to the development of the C-shape phenotype in *EPCAM*-KD cells (Supplementary Fig. 1). The difference between the proportion of C-shaped cells observed in this study (50%) and that observed after *EPCAM*-KD (30–40%) is probably due to the high doses of blebbistatin and Y-27632 they used, causing a massive inhibition of myosin-II. In *EPCAM*-KD cells, the loss of GTP-RhoA turnover leads to persistent RhoA signaling in the cell cortex, causing an abnormal continuous actomyosin contractility in the dorsal domain. As a result, protrusion stiffness remains high in *EPCAM*-KD cells (Fig. 3a–f), suggesting an increase of membrane tension. Literature has reported that treatment with Y-27632 or blebbistatin softens the actin cortex^116,117^. Along these lines, treatment with lose dose of Y-27632 or blebbistatin, which partially restores cell organization in *EPCAM*-KD cells (Fig. 5e, f), could lead to a softer cortex in the protrusion, more prone to cell deformation. We then hypothesize that the remodeling of the active RhoA zone would take place during the transition between phases P1 and P2 and/or at the onset of the P2 phase. The dynamic signaling mechanism of RhoA described above would support the earlier spreading model outlined by Sheetz and colleagues, and we propose that the exit of RhoA from the endosomal recycling compartments could constitute a spatio-temporal signal in this sequence of events. RhoA trafficking could indeed provide a rapid response to high membrane tension and thus contribute to the progression through the late phases of spreading. Another explanation would depend on the activity of actomyosin itself. Several studies have reported that pulsatile contractions occur at the medio-apical and junctional pools of actomyosin to facilitate cell shape changes and tissue morphogenesis in various species^39,118^. The expression of a constitutively active myosin-II phospho-mutant disrupts pulsatile contractility and delays tissue invagination during gastrulation in Drosophila^119^. Similarly, we could propose that the persistence of GTP-RhoA in the cell cortex could hinder the generation of effective contractility to ensure the transition from RFs and CAs to VSFs.

In contrast to the commonly accepted view of RhoA cycle at the plasma membrane, our work places a large pool of active RhoA in trafficking pathways, raising the following question: why the need for endosomal GTP-RhoA progression? Several hypotheses could be envisioned. This could allow fine tuning of GTP-RhoA distribution during spreading and migration in a constantly changing cell shape environment. At another level, the transit of GTP-RhoA through the endosomal compartments could help regulate its activation/inactivation cycle^39,40^. This would imply that RhoA regulators or effectors are located in distinct intracellular compartments. The cycle of RhoA between its active and inactive state is triggered by the sequential action of its guanine nucleotide exchange factors (GEFs), which promote GTP loading, and GTPase-activating proteins (GAPs) which favor GTP hydrolysis^31,36,37^. The intracellular traffic of RhoA could then be used to direct GTP-RhoA to other intracellular or plasma membrane domains to meet regulators or effectors. In fact, recent works and reviews have proposed that the RhoA activity patterns would be dependent on the subcellular distribution of GEFs and GAPs in function-oriented domains, rather than on the GTPase’s location itself^120^. More than 80 Rho GEFs and GAPs have been reported in the human genome^121^, suggesting that Rho GTPase cycles would be more specifically regulated than by a simple ON–OFF switch. Depending on the traffic pathway used to remodel the active RhoA zone, different combinations of GEFs and GAPs would guarantee an extreme precision for the spatio-temporal control of RhoA’s activity. The careful characterization of the subcellular patterning of GEFs and GAPs would be laborious but still remains an important subject of study. An alternative view is that endosomal trafficking could regulate RhoA signaling by redirecting GTP-RhoA away from the actomyosin cable region and/or prevent GTP-RhoA from encountering its effectors ROCK1, mDia, and MLCP. In this context, it will be necessary to analyze in the future whether there is a functional correlation between intracellular compartment positioning, such as recycling endosomes, RhoA signaling and activity, and cell organization.

**Fig. 9:**
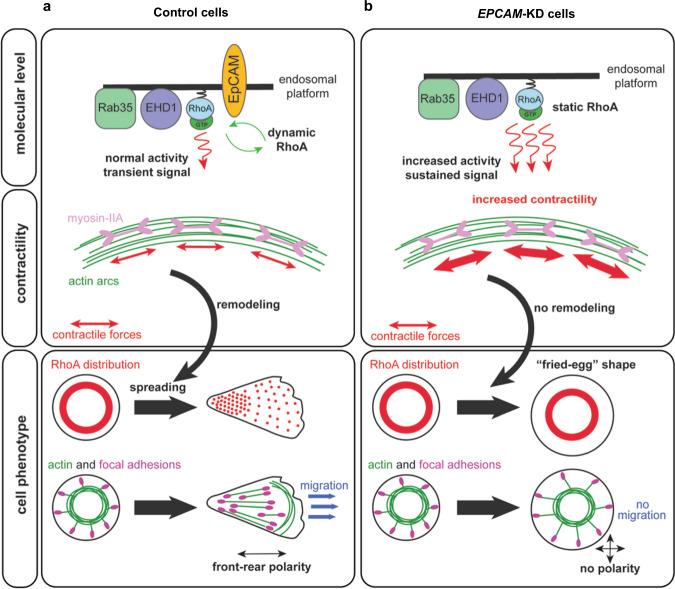
Scheme depicting the proposed model of active RhoA dynamics in control and *EPCAM*-KD cells during spreading. **a** In control cells, active RhoA (RhoA-GTP) dynamics are promoted by EpCAM, to and from the Rab35^+^/EHD1^+^ endosomal platform. The resulting transient signal induces normal myosin-II-dependent contractility at the level of the transverse arcs during spreading. At the cellular level, dynamic RhoA-GTP can be remodeled in a front–rear gradient as the cell spreads, participating to the acquisition of front–rear polarity. Correct contractility at the levels of the transverse arcs allows the formation of ventral stress fibers and proper actomyosin cytoskeleton reorganization to promote epithelial cell migration. **b** In *EPCAM*-KD cells, active RhoA is blocked in the endosomal platform preventing the remodeling necessary for correct spreading, symmetry breaking, and polarity establishment. RhoA-sustained activity increases myosin-II contractility at the transverse arcs level, which hinders the formation of ventral stress fibers. Active RhoA also increases formin activity, producing longer dorsal fibers in *EPCAM*-KD cells. The absence of active RhoA and actomyosin cytoskeleton remodeling impedes symmetry breaking, giving *EPCAM*-KD cells a characteristic unpolarized fried-egg shape and preventing efficient cell migration.

In light of the data presented in this study, we propose a model where EpCAM-mediated endosomal remodeling allows local modulation of RhoA signaling in space and time during epithelial spreading (Fig. 9). The canonical picture states that active RhoA is restricted to the plasma membrane^31,122^. While trafficking of transmembrane receptors, such as integrins or cadherins, has been scrutinized in the context of cell migration and cancer metastasis^123–125^, the link between traffic and Rho GTPases is now coming to the forefront. Our data support the idea that endosomes behave as platforms for Rho GTPase activation and spatio-temporal regulation^126^. Recent studies have described the presence of active Rho GTPases on intracellular membranes and argue in favor of Rho signaling originating from the endosomal network^126^. Lately, Vassilev et al. have shown that, in neural crest cells, RhoA trafficking is mediated by the Rab11-positive recycling pathway^91^. However, our findings demonstrate that, although a small proportion of active RhoA is indeed carried by Rab11-positive compartments (Supplementary Fig. 9b, c), the major endosomal pathway for RhoA is actually mediated by the Rab35/EHD1-positive compartments in epithelial cells (Fig. 7). In cortical endosomes, Rab35 and EHD1 control the fast recycling process, which acts in parallel to the slower canonical Rab11 pathway^99^. Whereas Rab35 operates during the early steps of endosomal recycling, EHD1 is required for late endosomal scission events^95,99,127^. Even though the functions of Rab35 in cell migration and adhesion vary according to cell types or migration assays, it emerges as a central component during cancer progression for receptor presentation, actin dynamics, and cell polarity^128,129^. A link between Rab35 and RhoA has even been mentioned previously, although not clearly demonstrated nor with any apparent impact^130^. At this stage, we can only speculate that distinct endosomal compartments could constitute cortical reservoirs of GTP-RhoA that cells would differentially use to reorganize the contractile network and generate forces in response to various situations or external cues. We realize that, for now, our study provides only a small window on the spatio-temporal regulation of contractility in epithelial cells. A more complete view of the upstream regulatory mechanisms and traffic pathways involved in the regulation of RhoA activity will deserve future in-depth analyses. Moreover, as numerous reports have pointed out the importance of RhoA in maintaining myosin-II activity and junctional integrity^131,132^, testing whether a similar endocytic mechanism occurs at cell contacts would be of great interest in the future.

In summary, our results show that endosomal trafficking is a central mechanism of spatio-temporal control of RhoA activity during SF formation and cell polarity acquisition in epithelial cells. We also provide a mechanistic understanding with the characterization of EpCAM-mediated active RhoA turnover through the cortical endosomes.

## Methods

### Cell culture

Caco2, MDCK, U2OS, and HeLa cells, originally acquired from ATCC, were kindly provided by Dr. S. Robine (Curie Institute, Paris), Pr. R. Jacob (Philipps University, Marburg), and Dr. V. Doye (Institut Jacques Monod, Paris), respectively. Caco2 and MDCK cells were routinely grown in Dulbecco’s modified Eagle’s medium (DMEM) 4.5 g/l glucose supplemented with 20% (Caco2 cells) or 10% fetal bovine serum (FBS) and 1% penicillin–streptomycin (Gibco, Thermo Fischer Scientific, Waltham, MA, USA) for a maximum of 9 passages. The culture medium was renewed every 2 days. For all experiments, cells were plated on collagen-coated substrates, obtained by adsorption of collagen I (Sigma-Aldrich) that was incubated at room temperature (RT) for 1 h at 100 μg/ml in 0.02 N acetic acid, and washed with phosphate-buffered saline (PBS) before cell seeding. *EPCAM*-KD was carried out by lentiviral delivery of shRNA constructs directed against human *EPCAM* designed and cloned into the lentiviral pLKO.1 puromycin-resistant vector Mission shRNA lentiviral transduction particle (Sigma-Aldrich). Control Caco2 clones (shNT) were generated using pLKO.1-puro non-target shRNA control transduction particles (Sigma-Aldrich). shRNA-resistant *EPCAM* sequence was provided by Invitrogen and cloned into a pEGFP-N1 backbone. sh*EPCAM*#1-resistant Caco-2 clones were generated by transfection with Lipofectamine 2000 (Thermo Fisher Scientific) according to the manufacturer’s instructions. Clone selection was performed in DMEM supplemented with 20% FBS, 10% penicillin/streptomycin, 2 µg/ml puromycin, and 0.5 mg/ml geneticin (Life Technologies, Paisley, UK). Plasmid and siRNA transient transfections were performed using Lipofectamine 2000. ACTN4 silencing was carried out using two siRNA targeting human ACTN4 mRNA, from Sigma-Aldrich. *EPCAM* reduction in MDCK cells was carried out using two siRNAs targeting dog *EPCAM* mRNA, purchased from Invitrogen. A detailed list of the primers, shRNA, and siRNA constructs is provided in Supplementary Table 1.

EpCAM-GFP was purchased from Origene (NM_002354, CAT# RG201989). Rab11-dominant-negative mutant was purchased from Addgene (#12678). AHPH-GFP, AHPH-mCherry, AHPH^A740D^, ROCK1-GBD-GFP, and mDia-GBD-GFP constructs were a kind gift from Dr. A. Yap (Brisbane University, Australia). Rho- GFP wt and mutants were a gift from Dr. Anne Blangy (CRBM, Montpellier, France). Rab4-mCherry, Rab7-GFP, caveolin-1-RFP, and Rab11-GFP constructs were a gift from Dr. C. Wunder (Curie Institute, France). Rab5-mCherry and Rab8-mCherry were a gift from Dr. G. Montagnac (Gustave Roussy Institute, France). EHD1-GFP and EHD1-G65R-GFP were a gift from Dr. S. Caplan (University of Nebraska Medical Center, NE, USA). Rab35-RFP and Rab35-S22N-RFP were provided by Dr. A. Echard (Pasteur Institute, Paris, France). LifeAct-GFP was from Addgene.

### Antibodies and reagents

Rabbit polyclonal antibody directed against EpCAM (#ab71916, immunofluorescence (IF) dilution, 1:100) and mouse monoclonal antibody directed against zyxin (#ab58210, IF dilution 1:100) were from Abcam. Mouse monoclonal antibodies directed against paxillin (clone 5H11, #05-41A, IF dilution, 1:100) and talin (clone TA205, #05-385, IF dilution, 1:100) were from Merck Millipore. Rat monoclonal antibody against activated β1-integrin, against EEA1, or against LAMP-1 were from BD Biosciences (#553715, IF dilution: 1/100; #610457, IF dilution, 1:100; #555798, IF dilution: 1:100). Rabbit polyclonal antibody against vinculin (# V4139, IF dilution, 1:100) and mouse monoclonal against α-tubulin (clone DM1A, #T9026) were from Sigma-Aldrich. Rabbit polyclonal antibody directed against MLC2 (#3672, WB dilution 1:1000) and P-MLC2 (T18/S19, #3674S IF dilution 1:100, WB dilution 1:500) were from Cell Signaling (Danvers, MA, USA). Rabbit monoclonal antibody directed against α4-actinin was from Invitrogen (#42-1400, IF dilution, 1:100). Monoclonal antibody directed against GAPDH (#60004-1-Ig, clone 1E6D9, WB dilution, 1:500) was from Proteintech (Chicago, IL, USA). Rabbit polyclonal antibody directed against Myosin-IIA (#909801, IF dilution 1:100) was from Biolegend (Princeton, NJ, USA). Phalloidin-Alexa488, 568, or 647 were from Invitrogen (#A12379, #A12380, and #A22287, respectively). Alexa Fluor 488-linked anti-mouse, anti-rat, and anti-rabbit antibodies (#A-11001, #A-11006, and #A-11008, respectively) and Alexa Fluor 568-linked anti-mouse and anti-rabbit antibody (#A-11001 and #A-11011, respectively) were from Invitrogen. Horseradish peroxidase (HRP)-linked anti-mouse (#A9044) and anti-rabbit (#A0545) antibodies were from Sigma-Aldrich. Blebbistatin, Y-27632, CK666, ML-7, and SMIFH2 were from Sigma-Aldrich (Saint Louis, MO, USA). NSC-23766 was from Tocris (Bio-Techne, France), and CN03 was from Cytoskeleton (Denver, CO, USA).

### Biochemical analysis

For WB, cell lysates were prepared 1 or 21 days after plating for protein detection in single cells or polarized monolayer, respectively. Cells were lysed for 30 min using the following lysis buffer: 50 mM Tris/HCl pH 8.0, 150 mM NaCl, 1 mM dithiothreitol, 0.5% NP-40, 1% Triton X-100, 1 mM EGTA, 1 mM EDTA, with complete protease inhibitor cocktail and phosphatase inhibitor PhosSTOP (Roche, Basel, Switzerland). Insoluble debris were removed by centrifugation at 13,000 × *g* for 15 min. Total protein content was measured by Bradford assay (Biorad). For each condition, 50 mg of proteins were loaded per well in Novex Tris-Glycine pre-cast gels (Thermo Fischer Scientific) and transferred on nitrocellulose membranes using iBlot Dry blotting system (Thermo Fischer Scientific). Proteins were detected with either HRP-linked goat anti-mouse IgG antibody (dilution 1:10,000; Sigma-Aldrich) or HRP-linked donkey anti-rabbit IgG antibody (dilution 1:10,000, GE Healthcare, Buckinghamshire, UK), and SuperSigna West Femto Maximum Sensitivity Substrate (Thermo Fischer Scientific) and visualized on ImageQuant LAS4000 (GE-Healthcare) and ChemiDoc and Image Lab Touch (2.3.0.07) software (Biorad). Signal quantification was performed using the Fiji software. Uncropped and unprocessed scans of WBs are presented in Supplementary Figs. 11 and 12.

For immunoprecipitation, cells were lysed as described above. Lysates were precleared with protein A–Sepharose beads (Sigma-Aldrich) for 1 h, incubated with antibodies overnight at 4 °C, and incubated with newly prepared protein A–Sepharose beads the next day for 2 h. The beads were washed three times with the lysis buffer. Precipitates were separated by sodium dodecyl sulfate-polyacrylamide gel electrophoresis and analyzed by immunoblotting.

### Immunostaining

Cells were fixed using 4% paraformaldehyde for 15 min, then permeabilized using 0.02% saponin solution in PBS for 20 min. In all, 0.02% saponin/1% BSA solution was used for a 30-min blocking step, before proceeding to incubation with the primary antibody at 4 °C overnight. The next day, secondary antibody was added after 3 washing steps in PBS and left to incubate for 2 h at RT. Except for structured illumination microscopic (SIM) analysis where Vectashield medium was used, all staining were mounted in Mowiol.

### Live imaging

Live cell spreading and migratory assays were performed with the Biostation (Nikon, Tokyo, Japan) using the ×20 objective. Time-lapse images were taken every 10 min for 2–4 h. Cell were treated with mitomycin C (10 μg/ml; Sigma-Aldrich) for 1 h to prevent division, before seeding on collagen-coated glass bottom fluorodishes (#FD35-100, World Precision Instruments, Sarasota, FL, USA). Cell track measurements and graphs were obtained using MATLAB (Mathworks, Natick, MA, USA).

Actin dynamics experiments were performed using an inverted DMI8 Leica microscope equipped with a CSU-W1 spinning disk head (Yokogawa–Andor), using a ×100 1.4 NA oil objective and Metamorph (7.10.1.161) software (Molecular Devices). Images were acquired every 5 or 12 min for 2–4 h. Active RhoA and EHD1 dynamics were followed on the same microscope, for 2 min with a 1-s frame rate. For the analysis of AHPH probe and EHD1-positive compartment contact time, AHPH-mCherry vesicles were manually tracked and EHD1-GFP contact length was manually assessed.

Color-coded *t*-projection of AHPH-mCherry were generated from spinning disc acquisitions^133^. Ten frame *t*-stack were selected from time-lapse series. The first image (t0) was false-colored green, the last image (t9) was false-colored in blue, and the intervening time points (t2–8) were submitted to *t*-projection and shown in red (*t*-projection), and images were merged.

### Structured illumination microscopy

3D-SIM was performed on a Zeiss Elyra Microscope coupled to an optovar 1.6, ×63 objective, and a camera EM CCD Andor SIM. During *z*-stack acquisition, five rotations were applied. Deconvoluted structured illumination images were generated by the Zen software, and images were merged in ImageJ.

### Atomic force microscopy

Cells were cultured in DMEM, 20% FBS, 1× penicillin–streptomycin, and 2 μg/ml puromycin. Plastic petri dishes (TPP, Switzerland) were incubated in 100 μg/ml rat tail collagen I (Gibco A10483-01) in 0.1% acetic acid at 4 °C overnight on a 60 rpm shaker. Cells were seeded at low density and allowed to adhere at least 5 h. AFM nanoindentation experiments were performed with a Nanowizard 4 (JPK Instruments, Germany) in Quantitative Imaging™ mode. The imaging buffer was Leibovitz L-15 medium supplemented with 20% FBS and 1× penicillin-streptomycin and experiments were performed at 37 °C with a petri dish heater. PFQNM-LC-A-CAL cantilevers (Bruker, USA) were used; the tip was parabolic (Hertz model) at the apex with *r* = 35 nm as determined by scanning electron microscopy (see Supplementary Fig. 3a, b). Electron microscopy was performed using a JEOL JSM-6010LV microscope. The probe spring constant was provided by the manufacturer (*k* = 0.068 N/m), and the optical lever sensitivity was determined by the thermal tuning method. Force–indentation curves were collected with 100 μm/s probe velocity, 400 pN trigger force, and variable indentation–retraction distance (scanning frequency) over a (60 μm)^2^ area with 128 × 128-pixel resolution (each cell scan lasted ~10 min, the fast axis was horizontal in images shown). Data were analyzed using a custom-built MATLAB program adapted from previous work^134,135^. This MATLAB-based custom program for AFM analyses^136^ is available at https://github.com/bryantdoss/matlab-afm-indentation (10.5281/zenodo.4543281). Force–indentation curves were fit by inverting the power–law of the force–indentation curve and then fitting this to a linear slope and *x*-intercept to determine the apparent elastic modulus and contact point, respectively^134^; indentation depths up to 800 nm were analyzed and the Poisson’s ratio is assumed to be 0.5. All force–indentation curves were first corrected for virtual deflection and initially fit using the Hertz model for the contact point and an initial modulus without any correction for the rigid substrate. Very stiff regions (>65 kPa) were assumed to be the plastic substrate, and the height of all locations was determined by linear flattening of the contact points on plastic regions. Once the height of each pixel was known, force–indentation curves were fit again for the apparent elastic modulus to correct for the rigid substrate with the Hertz model modified for a thin sample adhered to an infinitely rigid substrate^74^. Cells were masked by excluding regions <100 nm in height or >65 kPa stiffness, and curves that did not have an identifiable baseline were excluded from subsequent analysis.

### Traction force microscopy

Soft polydimethylsiloxane substrates of 15 kPa rigidity containing red 200 nm fluorescent beads (Life technologies) were prepared by mixing CY52-276 kit components (Dow Corning Toray) at 1:1 ratio and letting it spread and cure on glass bottom fluorodishes overnight. Collagen I (Sigma-Aldrich) was adsorbed on the surface on the substrate as described above. Cells were plated on the substrate and imaged for 22–24 h, at a 6-min interval in the Biostation using the ×20 objective. Images were aligned to compensate for experimental drift before analysis of the beads displacement was performed, using Particle Imaging Velocimetry script in MATLAB. From the displacement data, Fourier transform traction cytometry plugin in ImageJ (available at https://sites.google.com/site/qingzongtseng/tfm) was used to estimate the traction forces exerted on the substrate. The total traction force exerted by cells was calculated by summing the magnitudes of the traction vectors under and near the cell of interest and multiplying by the area covered by those vectors.

### FRET analyses

The FRET probe Raichu-1502 (also named Raichu-RBD^81^) was transfected into Caco2 cells 48 h before the experiment. Spectral imaging was performed on a confocal LSM780 microscope (Zeiss, Zen software) with ×63/1.4NA plan apochromat oil-immersion objective. CFP was excited by the 458-nm laser line of an Argon laser, and emission was sampled at a spectral resolution of 9-nm within a 444–570-nm range. ImageJ was used to process images for analyses. FRET ratio was calculated as the ratio between the YFP and CFP signal.

### Statistical analysis

All statistical analyses were performed using Prism (GraphPad Software, San Diego, CA, USA, version 7.0). Unless otherwise stated, experiments were replicated three times independently and comparison between samples were done without Gaussian distribution assumption of the data, meaning comparisons were carried out using Mann–Whitney test for two conditions comparison, or Kruskal–Wallis test and Dunn’s multiple comparison tests for three and more conditions. *p* Values met the following criteria *p < 0.05, **p < 0.01, and ***p < 0.001.

Triple colocalization was analyzed from confocal stacks using a MATLAB-based custom program. Briefly, fluorescence was first segmented in each channel using local thresholding (Phansalkar method^137^ and a local two-dimensional median filter with user-defined 16 neighborhood size was applied to remove noise. Colocalization was then measured using the Manders split coefficients M1 and M2^138^ as: ${\mathrm{M}}1 = \frac{{\mathop {\sum }\nolimits_i {\mathrm{S}}1_{i,{\mathrm{coloc}}}}}{{\mathop {\sum }\nolimits_i {\mathrm{S}}1_i}}$ and ${\mathrm{M}}2 = \frac{{\mathop {\sum }\nolimits_j {\mathrm{S}}2_{j,{\mathrm{coloc}}}}}{{\mathop {\sum }\nolimits_j {\mathrm{S}}2_j}}$, where S1_*i*,coloc_ = S1_*i*_ if S1_*i*_ > 0 AND S2_*i*_ > 0 and S2_*j*,coloc_ = S2_*j*_ if S2_*j*_ > 0 AND S1_*j*_ > 0.

The MATLAB-based custom program for triple colocalization analysis and the standalone user interface^139^ are available at https://github.com/simondebeco/Colocalization-Analyzer (10.5281/zenodo.4501944).

### Reporting summary

Further information on research design is available in the Nature Research Reporting Summary linked to this article.

## Supplementary information

Supplementary Information

Description of Additional Supplementary Files

Supplementary Movie 1

Supplementary Movie 2

Supplementary Movie 3

Supplementary Movie 4

Supplementary Movie 5

Supplementary Movie 6

Supplementary Movie 7

Supplementary Movie 8

Supplementary Movie 9

Supplementary Movie 10

Supplementary Movie 11

Supplementary Movie 12

Reporting Summary

## Data Availability

The data supporting the findings of this study are available within the article and Supplementary Information. Uncropped and unprocessed scans of WBs are presented in Supplementary Figs. 11 and 12. Microscopic data are available from the corresponding author upon request due to their large file sizes. A reporting summary for this article is available as a Supplementary Information file. Source data are provided with this paper.

## Source data

Source Data

## Code availability

The codes of MATLAB-based custom program for AFM analyses^136^ and triple colocalization analyses^139^ are available on GitHub (https://github.com/bryantdoss/matlab-afm-indentation, 10.5281/zenodo.4543281 and https://github.com/simondebeco/Colocalization-Analyzer, 10.5281/zenodo.4501944).
