# Supplementary material for: EpCAM promotes endosomal modulation of the cortical RhoA zone for epithelial organization
Source: Nat Commun. 2021 Apr 13;12:2226. doi: 10.1038/s41467-021-22482-9 (PMC8044225; doi:10.1038/s41467-021-22482-9)
Supplement: Supplementary file 1 — Supplementary Information [file 41467_2021_22482_MOESM1_ESM.pdf]

Supplementary Materials for

EpCAM promotes endosomal modulation of the cortical RhoA zone for epithelial organization

Gaston Cécile[1], De Beco Simon[1], Doss Bryant[2], Pan Meng[2], Gauquelin Estelle[1], D'Alessandro Joseph[1], Lim Chwee Teck[2], Ladoux Benoit[1] and Delacour Delphine[1]*

[1]Cell adhesion and mechanics, Institut Jacques Monod, CNRS UMR7592, Paris Diderot University, 75205 Paris Cedex 13, France

[2]Mechanobiology Institute, T-lab, Singapore 117411, Singapore

* Corresponding author: delphine.delacour@ijm.fr

This PDF includes:

Supplementary Figures 1 to 12

Supplementary Tables 1 to 5

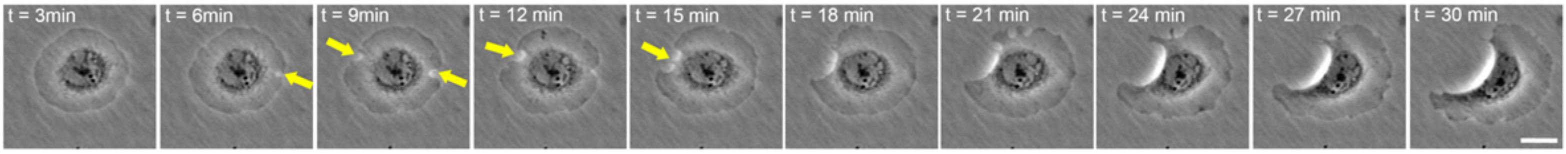

Gaston et al., Supplementary Figure 1

Supplementary Figure 1. Development of C-shaped cells in the absence of EpCAM. Phase contrast time-lapse of *EPCAM*-KD cells during cell spreading showing C-shape development. Yellow arrows point on symmetry breaking events. Scale bar, 5µm. Three independent experiments were carried out.

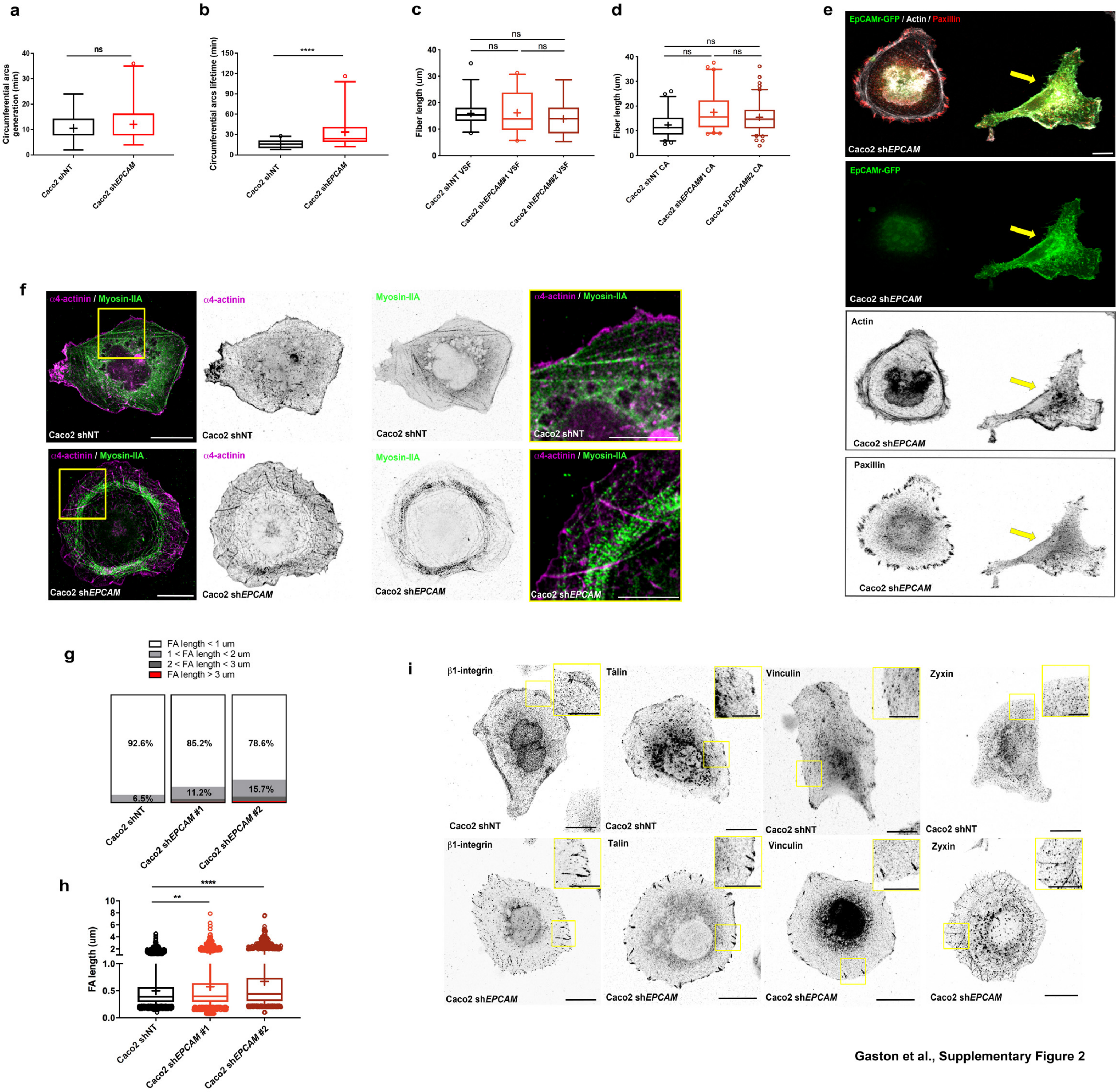

Gaston et al., Supplementary Figure 2

Supplementary Figure 2. Block of stress fiber maturation in the absence of EpCAM. a Analysis of the generation time (in min) of circumferential arcs (CAs) in control and *EPCAM*-KD cells. Mean generation time in shNT = 10.47± 5.46 (mean +/- SD), in sh*EPCAM* = 12.00±8.51. N (shNT) = 17 cells, N (sh*EPCAM*) = 24 cells. The box plot whiskers represent the 5-95 percentile confidence interval. The mean is displayed as a cross. Two-sided Mann-Whitney test, p = 0.925. **b** Analysis of CA lifetime (in min) in control and *EPCAM*-KD cells. Mean lifetime in shNT = 16.00± 5.92 (mean± SD), in sh*EPCAM* = 33.63± 24.86. N (shNT) = 22 cells, N (sh*EpCAM*) = 27. The box plot whiskers represent the 5-95 percentile confidence interval; the mean is displayed as a cross. Two-sided Mann-Whitney test, **** p<0.0001**.** **c** Statistical analysis of ventral stress fiber (VSF)' length (in µm) in control and *EPCAM*-KD cells. N (shNT) = 36 cells, N (sh*EpCAM#*1) = 35, N (sh*EpCAM#*2) = 43, n>16 VSFs for each condition. Kruzkal-Wallis test and Dunn's multiple comparison test, p>0.99. The box plot whiskers represent the 5-95 percentile confidence interval; the mean is displayed as a cross. **d** Statistical analysis of circumferential arc (CA)' length (in µm) in control and *EpCAM*-KD cells. N (shNT) = 29 cells, N (sh*EpCAM#*1) = 28, N (sh*EpCAM#*2) = 28. n> 58 CAs for each condition. Kruzkal-Wallis test and Dunn's multiple comparison test. ns, non-significant. The box plot whiskers represent the 5-95 percentile confidence interval; the mean is displayed as a cross. **e** Confocal analysis of actin (gray) and paxillin (red) in sh*EpCAM* cells after EpCAM rescue through the expression of an EpCAM-GFP construct resistant to the shRNA (EpCAMr-GFP, green). Projected confocal z-stacks are presented. Yellow arrow points to a cell where EpCAM expression was restored. Scale bar, 5µm. **f** Confocal analysis of the distribution of α4-actinin (magenta) and myosin-IIA (green) in control and *EpCAM*-KD cells. Scale bar, 5µm; insert scale bar, 2.5µm. **g** Analysis of the distribution of focal adhesions (FAs) according to their size in control and *EpCAM*-KD cells. N (shNT) = 31 cells, N (sh*EpCAM#*1) = 30, N (sh*EpCAM#*2) = 30. Chi-square test, **** p<0.0001. The exact number of FAs (and %) in each category is available in Supplementary table 5. **h** Analysis of the mean length of FAs in control and *EpCAM*-KD cells. Kruskal-Wallis test, p <0.0001 and Dunn's multiple comparison test, ** p = 0.0029, **** p < 0.0001. The box plot whiskers represent the 5-95 percentile confidence interval; the mean is displayed as a cross. N (shNT) = 31 cells, N (sh*EpCAM#*1) = 30, N (sh*EpCAM#*2) = 30. **i** Confocal analysis of the distribution of β1-integrin, talin, vinculin and zyxin in control and *EpCAM*-KD cells. Projected confocal z-stacks are presented. Scale bar, 5µm; insert scale bar, 2.5µm. For each experiment, three independent experiments were carried out. ns, non-significant difference.

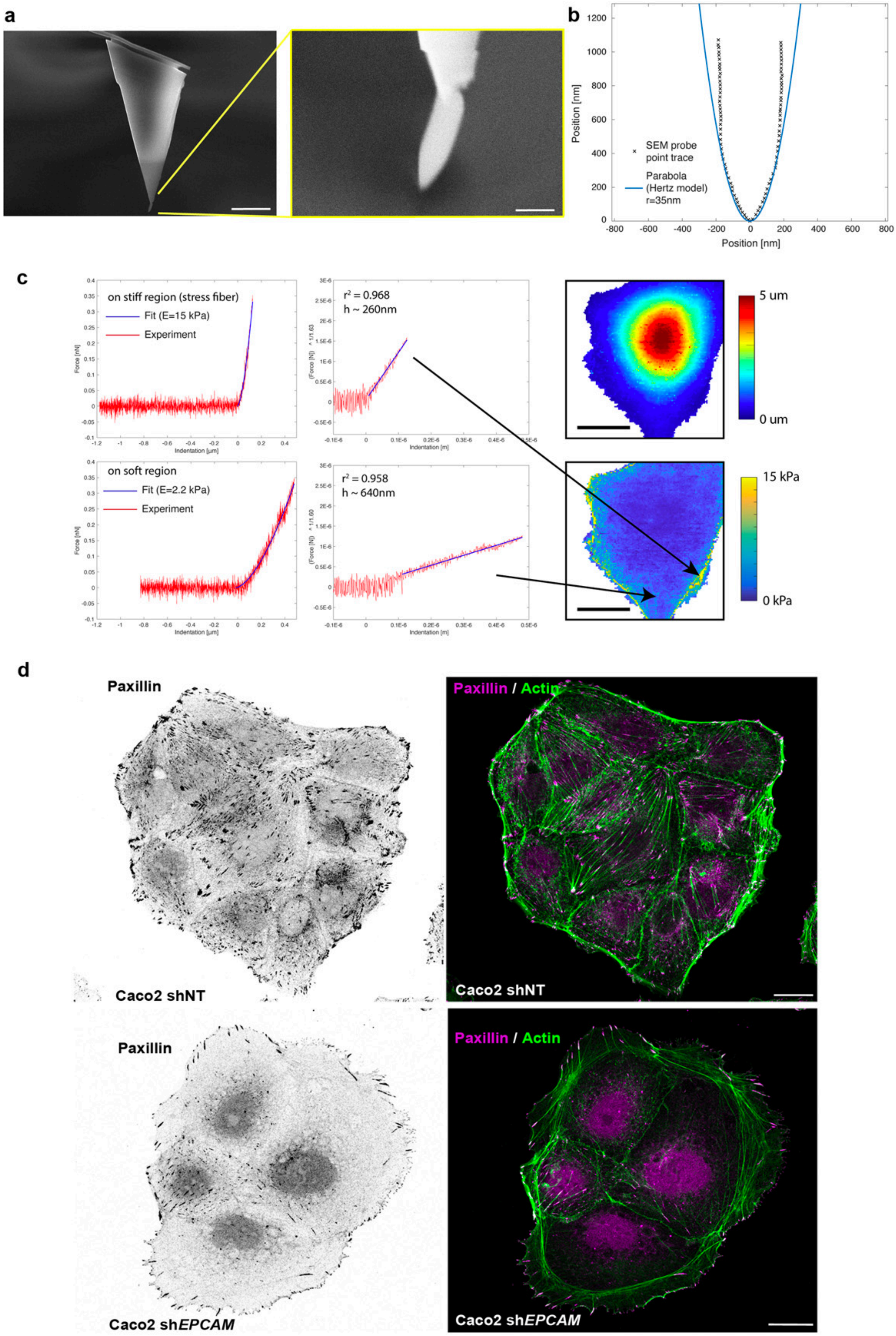

Gaston et al., Supplementary Figure 3

Supplementary Figure 3. Changes in cell stiffness and actin organization in the absence of EpCAM. a Scanning electron microscope (SEM) images of the PFQNM-LC-CAL probe used in the AFM experiments. Scale bar, 5µm; insert scale bar, 0.5µm. **b** Points on the SEM probe were traced (black) and plotted with a parabolic Hertz model indenter with 35 nm radius (blue). **c** Example raw force-indentation curves on stiff (top left) and soft (bottom left) regions, corresponding linearized force-indentation curves (center column), and corresponding topography (top right) and apparent elastic modulus maps (bottom right). Red shows experimental data aligned to the contact point and corrected for virtual deflection, and blue shows the fitted curve. Arrows from the center panels point to the corresponding pixel location in the apparent elasticity map. **d** Confocal analysis of actin (green) and paxillin (magenta) distribution in control and *EpCAM*-KD cell islands. Projected confocal z-stacks are presented. Scale bar, 5µm. Three independent experiments were carried out.

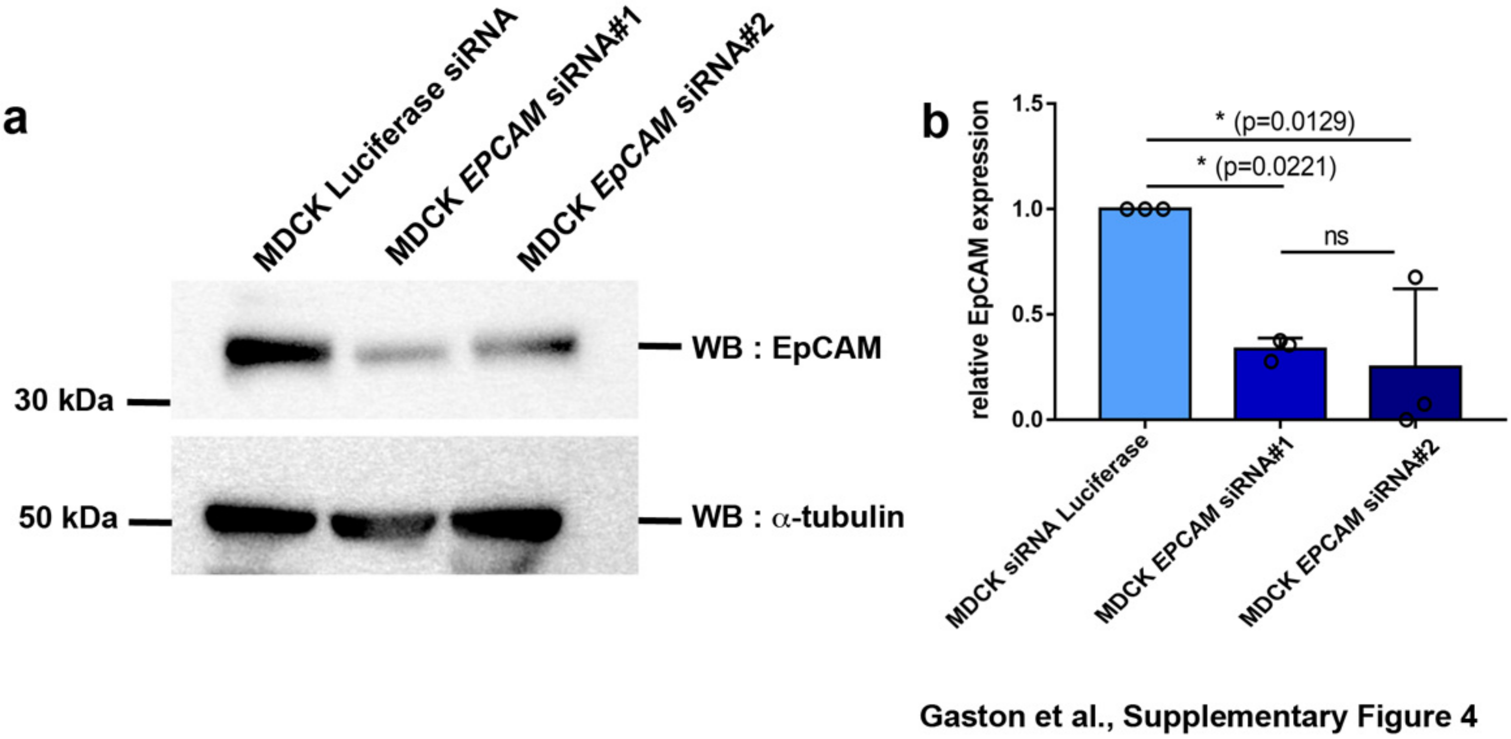

Supplementary Figure 4. *EPCAM* depletion in MDCK cells. a Western blot analysis of EpCAM expression in control or *EpCAM* siRNA-treated MDCK cells. α-tubulin was used as a loading control. **b** Statistical analysis of EpCAM expression in control and siRNA-treated MDCK cells. Data are mean ± SD. One-way ANOVA and Tukey's multiple comparison test, $p < 0.0104$; for Luciferase siRNA vs *EPCAM* siRNA#1, * $p = 0.0221$; for Luciferase siRNA vs *EPCAM* siRNA#2, * $p = 0.0129$. Extinction rates were 66% and 75% for siRNA#1 and siRNA#2, respectively. For each experiment, three independent experiments were carried out. ns, non-significant difference.

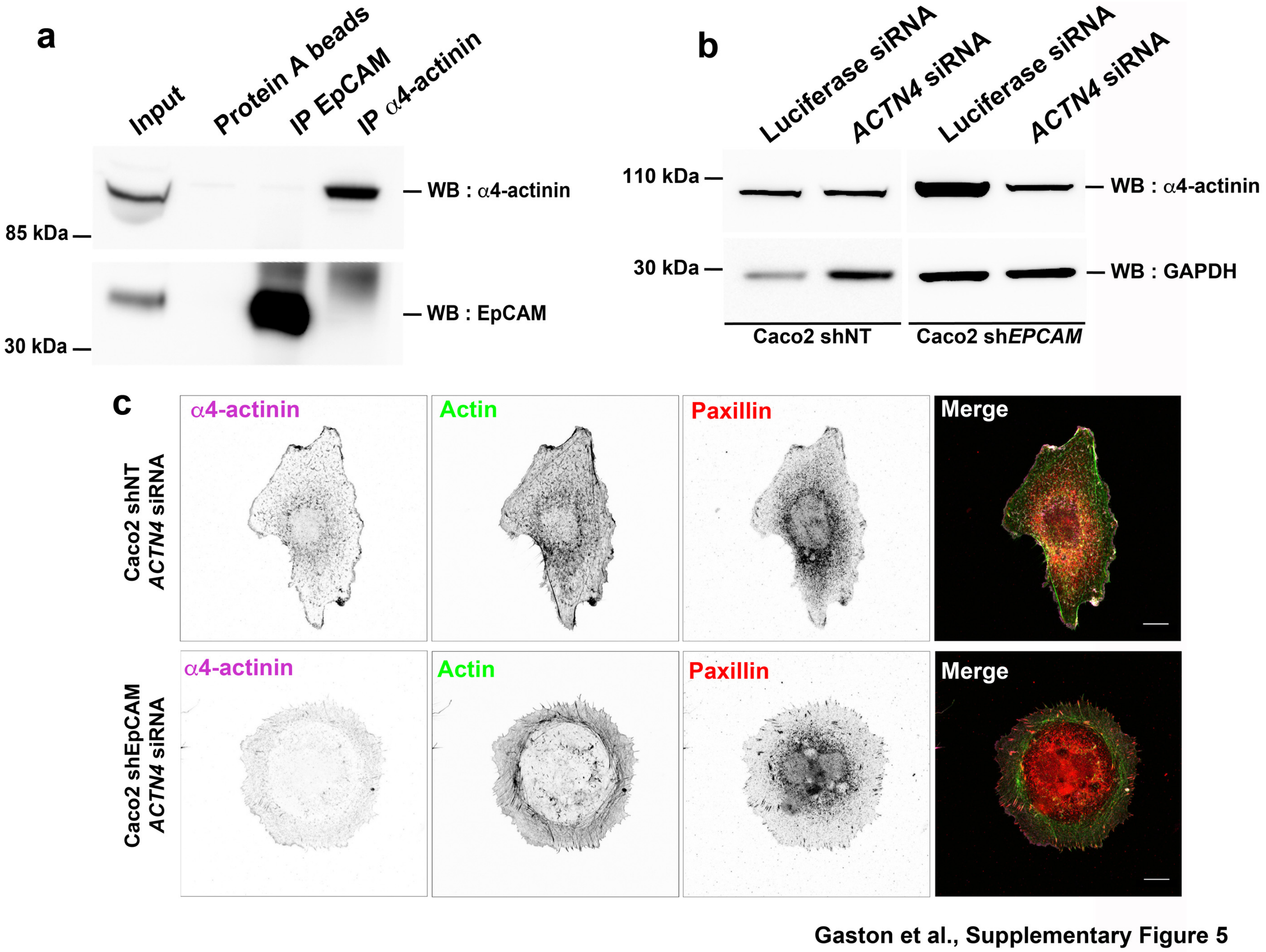

Supplementary Figure 5. α4-actinin is not involved in the EpCAM-dependent mechanism for SF organization. a Western blot detection of α4-actinin (upper panel) and EpCAM (lower panel) after immunoprecipitation of α4-actinin or EpCAM from Caco2 cell extracts. **b** Western blot analysis of α4-actinin expression in control (Luciferase siRNA) or α4-actinin-deprived *(ACTN4* siRNA) and *EPCAM*-KD cells. GAPDH was used as loading control. **c** Confocal analysis of α4-actinin (magenta), actin (green) and paxillin (red) distribution in *ACTN4*-KD control (upper panel) or in *ACTN4*-KD *EPCAM*-KD cells (lower panel). Projected confocal z-stacks are presented. Scale bar, 10μm. For each experiment, three independent experiments were carried out.

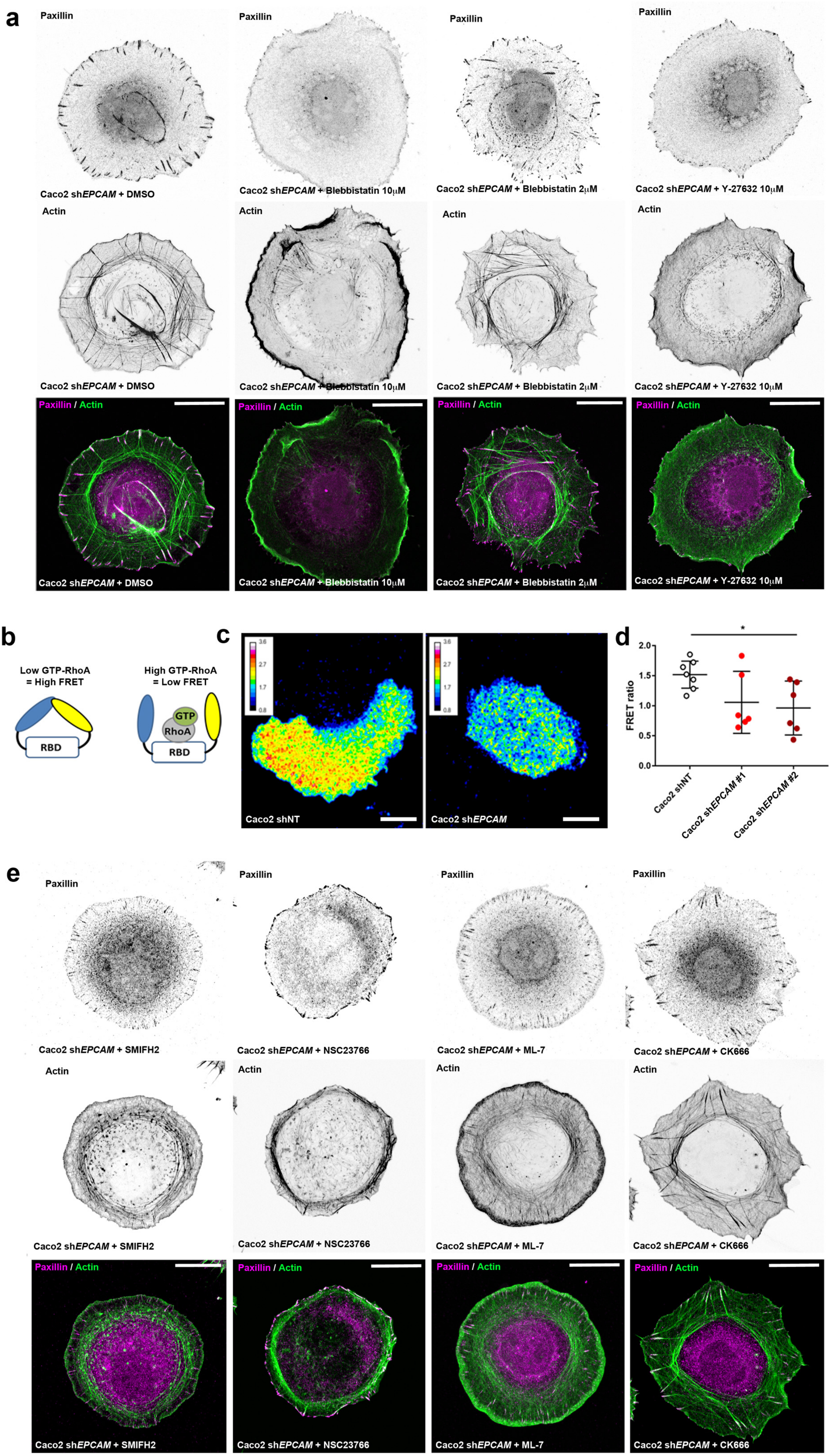

Gaston et al., Supplementary Figure 6

Supplementary Figure 6. Local hyperactivity of the actomyosin network is at the origin of SF and FA abnormalities in *EPCAM*-KD cells. a Confocal analysis of paxillin (magenta) and actin (green) in *EPCAM*-KD cells upon DMSO, blebbistatin 2 µM, blebbistatin 10 µM, Y-27632 10 µM treatment for 1 hour. Projected confocal z-stacks are presented. Scale bars, 5µm. Three independent experiments were carried out. **b** Scheme presenting the principle of the FRET probe to measure RhoA activity developed by Matsuda and colleagues (Yoshizaki et al., 2003). **c** FRET intensity maps were generated in control and *EPCAM*-KD cells with "16 colors" LUT table from ImageJ; color scale bar indicates the FRET intensity. Scale bars, 10µm. **d** Statistical analyses of FRET intensity in control and *EPCAM*-KD cells. FRET ratio in shNT cells = 1.52±0.24, sh*EPCAM*#1 cells = 1.06±0.49 and Caco2 sh*EPCAM*#2 cells = 0.96±0.43 (mean±SD). N (shNT) = 7 cells, N (sh*EPCAM*#1) = 6, N (sh*EPCAM*#2) = 6. One-way ANOVA test with Dunnett's multiple comparison test, *p=0.0456. Two independent experiments were carried out. **e** Confocal analysis of paxillin (magenta) and actin (green) in *EPCAM*-KD cells upon SMIFH2 2 nM, ML-7 10 µM, CK666 50 µM or NSC23766 50 µM treatment for 1 hour. Projected confocal z-stacks are presented. Scale bars, 5µm. Three independent experiments were carried out.

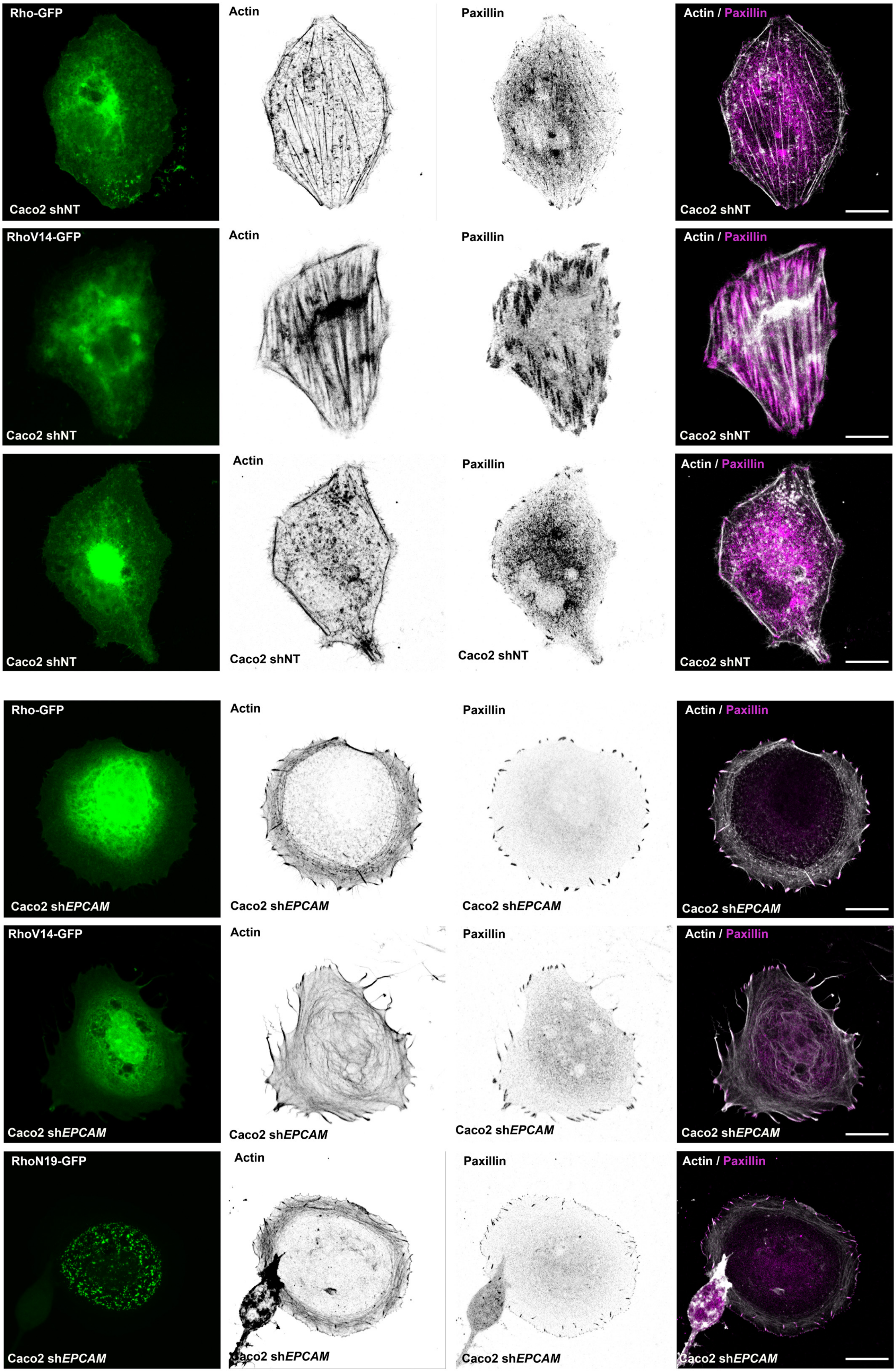

Gaston et al., Supplementary Figure 7

Supplementary Figure 7. The expression of Rho mutant forms in control cells only partially reproduced some aspects of the *EPCAM*-KD phenotype. Confocal analysis of paxillin (magenta) and actin (gray) in control and *EPCAM*-KD cells transfected with RhoA-GFP, RhoA V14-GFP or RhoA N19-GFP constructs (green). Projected confocal z-stacks are presented. Scale bars, 5µm. Three independent experiments were carried out.

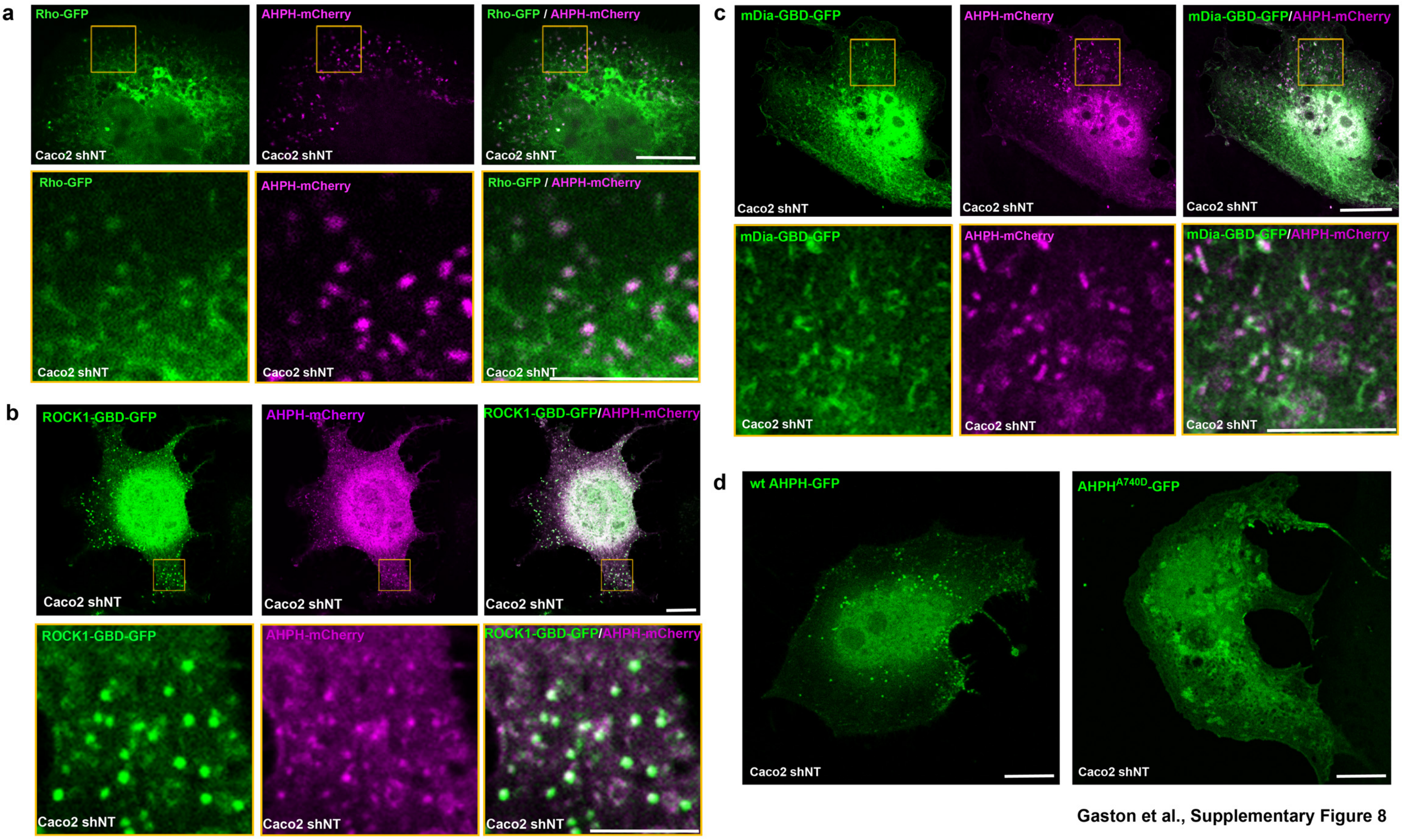

Supplementary Figure 8. Specificity of the tagged form of AHPH for GTP-RhoA signal. a-c Confocal analysis of total RhoA (Rho-GFP, green) (a), RhoA-GTP binding domain of ROCK1 (ROCK1-GBD-GFP, green) (b) or RhoA-GTP binding domain of mDia (mDia-GBD-GFP, green), together with GTP-RhoA (AHPH-mCherry, magenta) in control cells. **d** Confocal analysis of the localization of the wt AHPH-GFP and the mutant form $AHPH^{A740D}$-GFP in control cells. Scale bar, 5 µm; insert scale bar, 2.5 µm. For each experiment, three independent experiments were carried out.

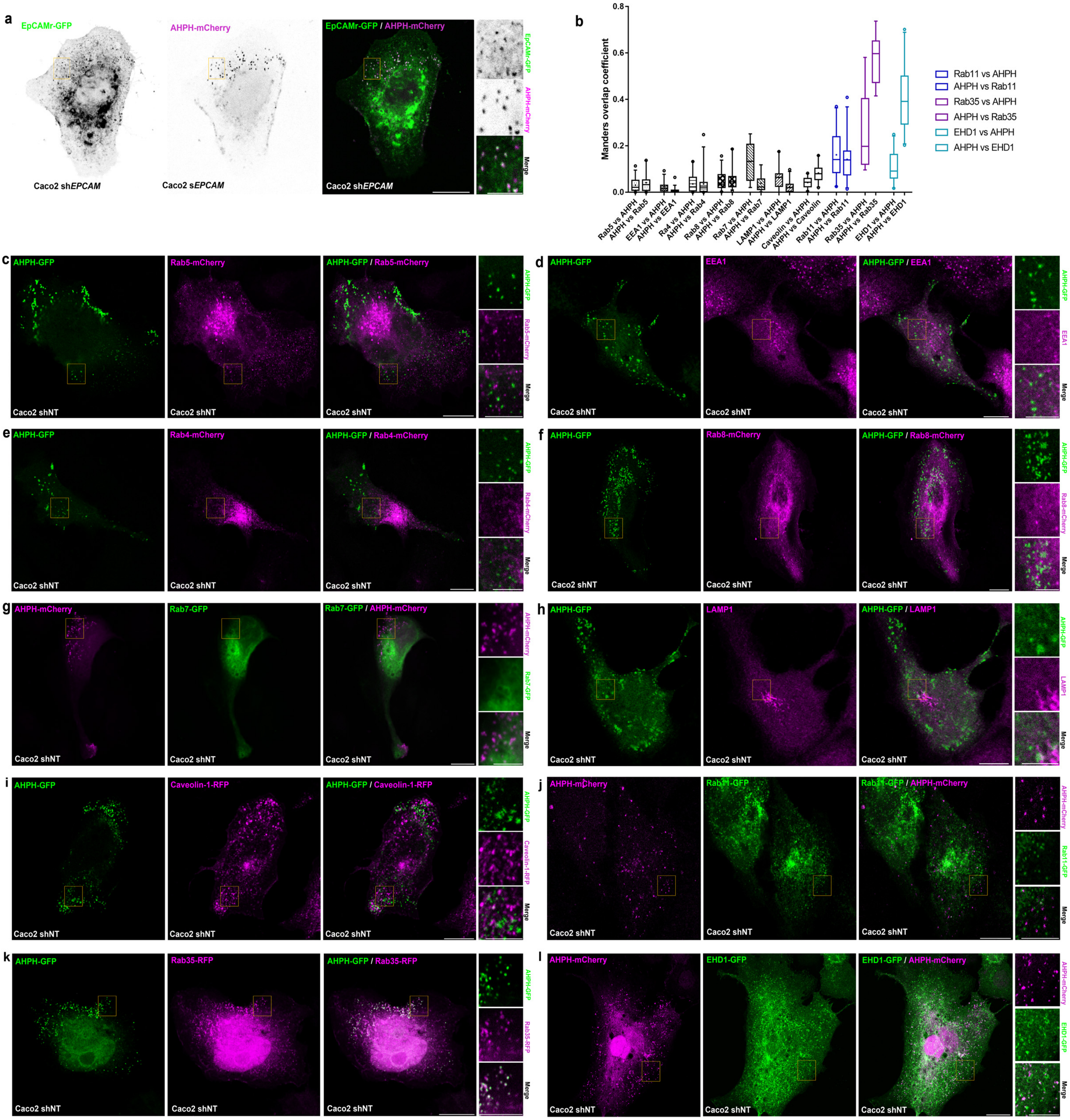

Gaston et al., Suppplementary Figure 9

Supplementary Figure 9. Intracellular localization of the AHPH probe. a Confocal analysis of AHPH-mCherry in *EPCAM*-KD cells after rescue with EpCAMr-GFP (green) transfection. Areas boxed in yellow are presented on the right. Scale bar, 5 µm; insert scale bar, 2 µm. **b** Quantification of the Manders overlap coefficient between AHPH-GFP versus different endosomal markers in control cells. N= 10 cells. Whiskers represent the 5-95 percentile confidence interval. The mean is displayed as a cross. **c-l** Confocal analysis of AHPH-GFP (green) and Rab5-mCherry (magenta) (c), AHPH-GFP (green) and EEA1 (magenta) (d), AHPH-GFP (green) and Rab4-mCherry (magenta) (e), AHPH-GFP (green) and Rab8-mCherry (magenta) (f), AHPH-mCherry (magenta) and Rab7-GFP (green) (g), AHPH-GFP (green) and LAMP1 (magenta) (h), AHPH-GFP (green) and caveolin-1-RFP (magenta) (i), AHPH-mCherry (magenta) and Rab11-GFP (green) (j), AHPH-GFP (green) and Rab35-RFP (magenta) (k) and AHPH-mCherry (magenta) and EHD1-GFP (green) (l) in control cells. Areas boxed in yellow are presented on the right. Scale bar, 5µm; insert scale bar, 2.5 µm. For each experiment, three independent experiments were carried out.

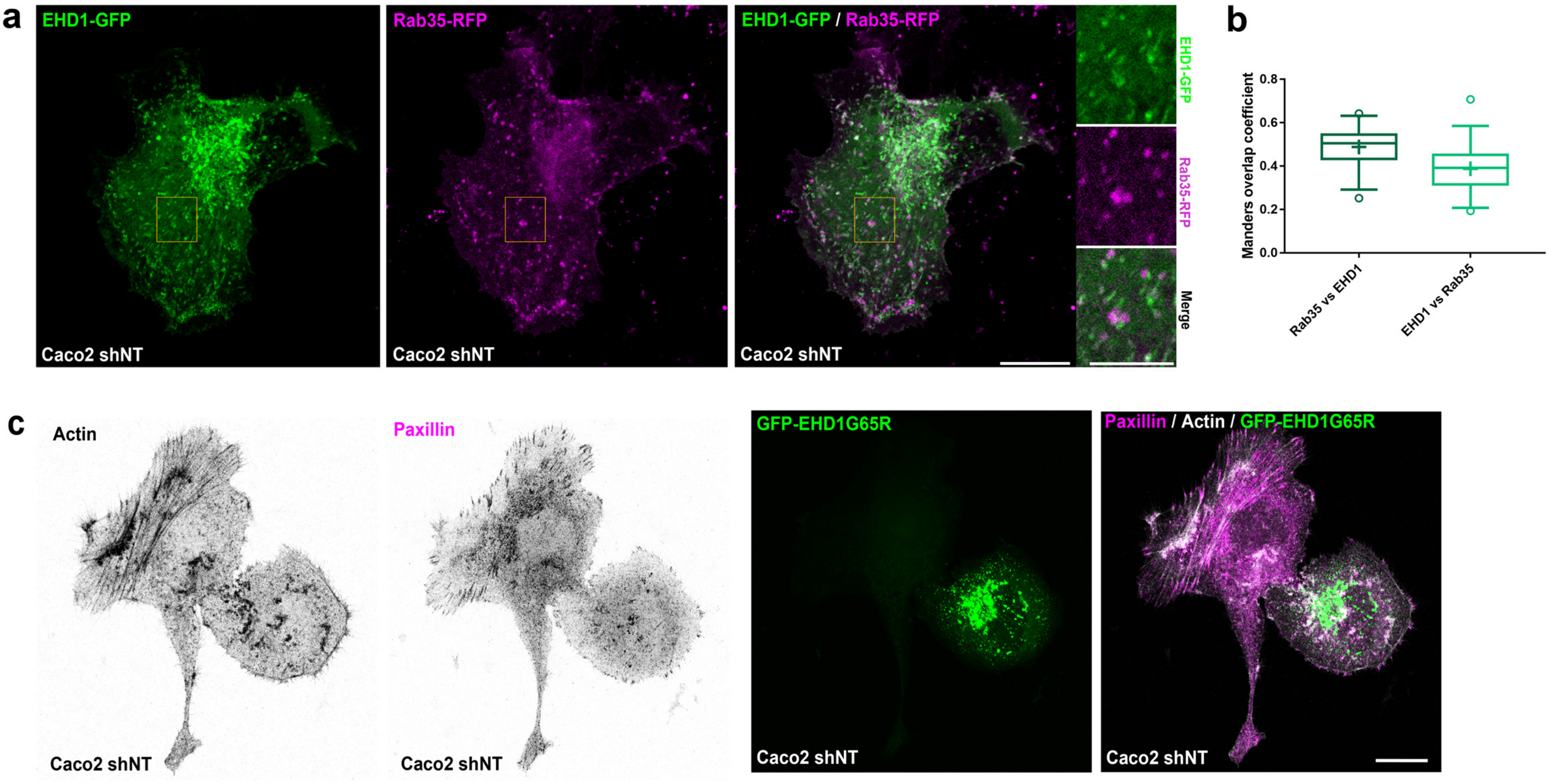

Gaston et al., Supplementary Figure 10

Supplementary Figure 10. Localization of EHD1 in Rab35-compartments, and impact of EHD1 on stress fiber organization. a Confocal analysis of EHD1-GFP and Rab35-RFP in control cells. Areas boxed in yellow are presented on the right. Scale bar, 5µm. **b** Quantification of the Manders overlap coefficient between EHD1-GFP versus Rab35-RFP in control cells. N= 10 cells. Whiskers represent the 5-95 percentile confidence interval. The mean is displayed as a cross. **c** Confocal analysis of actin (gray) and paxillin (magenta) distribution after EHD1G65R-GFP transfection in control cells. Scale bar, 5µm; insert scale bar, 2.5 µm. For each experiment, three independent experiments were carried out.

Figure 1a

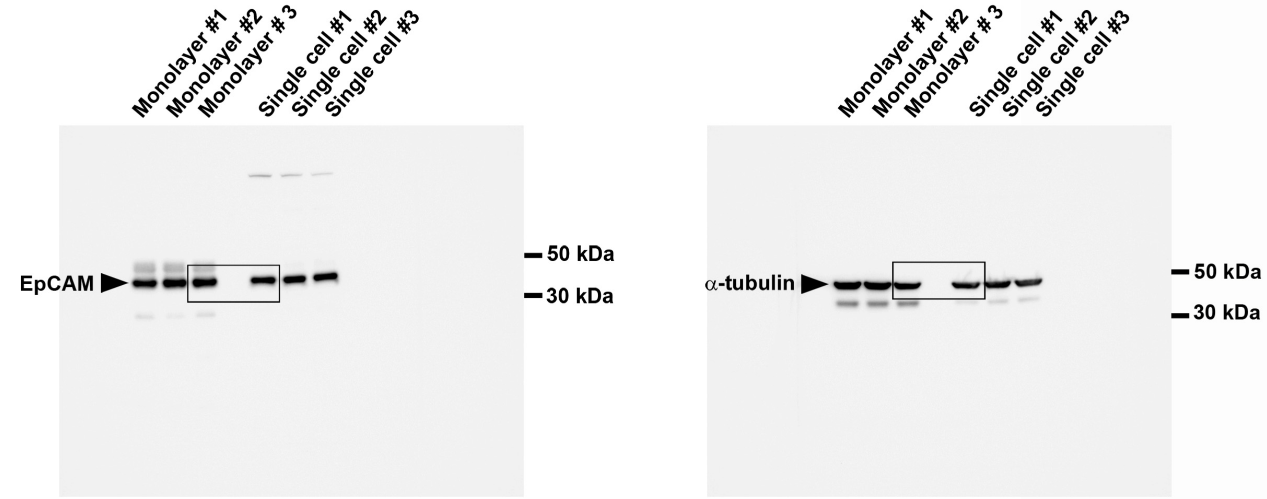

Figure 4a

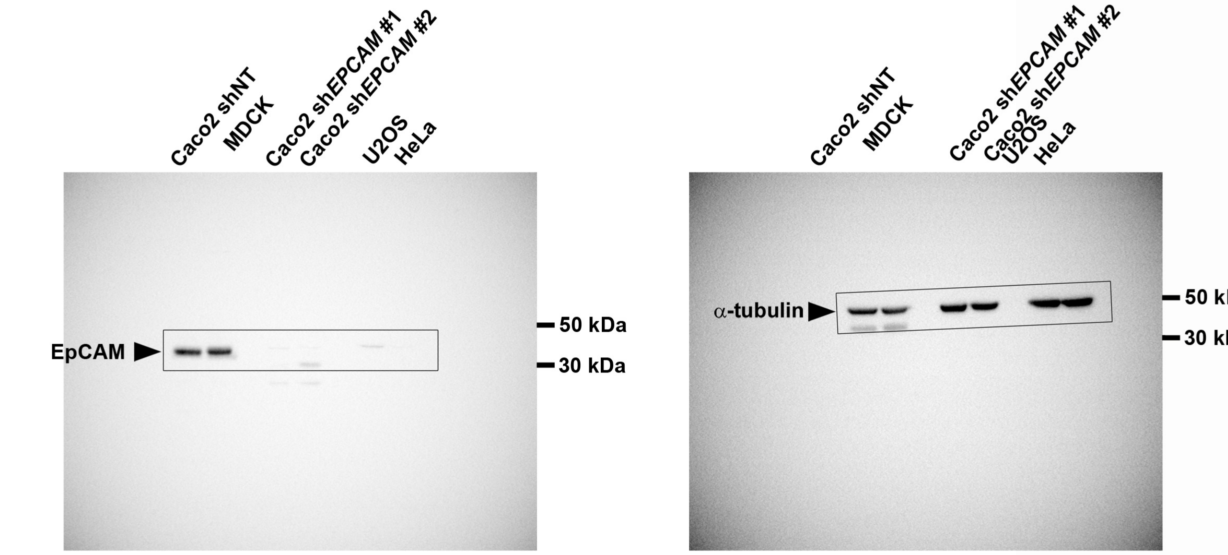

Figure 5c

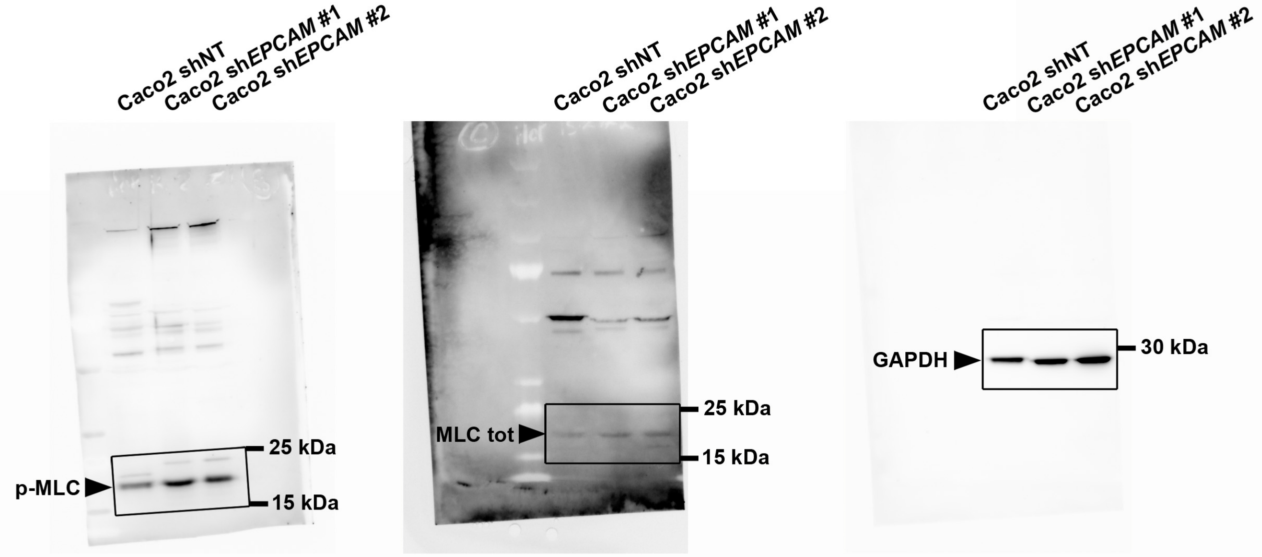

Gaston et al., Supplementary Figure 11

Supplementary Figures 11. Original western blots used in Figure 1a, 4a and 5c.

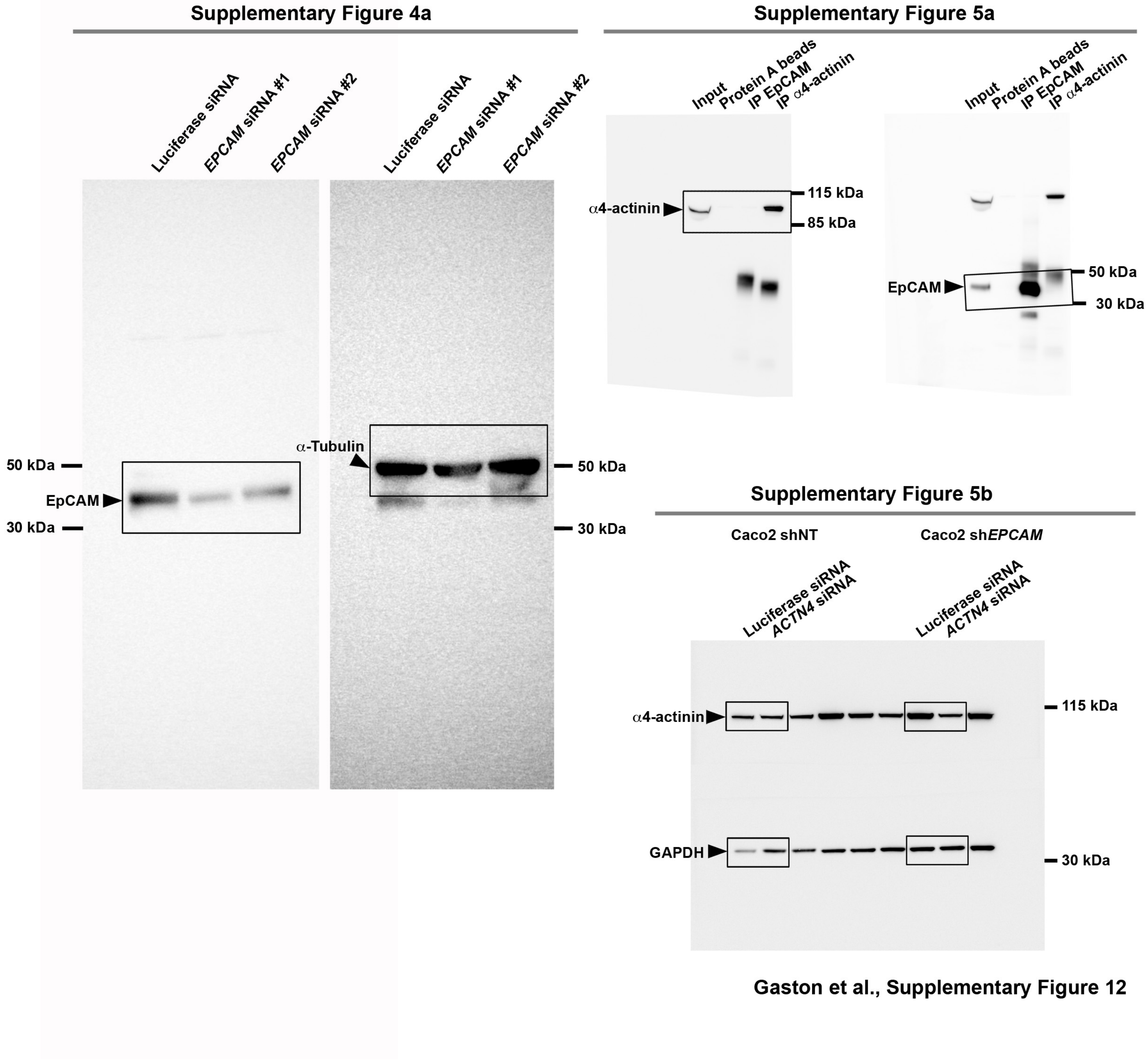

Supplementary Figures 12. Original western blots used in Supplementary Figures 4a, 5a and 5b.

Supplementary table 1. Detailed list of the primers, shRNA and siRNA constructs used in this study.

Product	Denomination	Sequence	Provider
shRNA constructs directed against human *EPCAM*	sh*EPCAM*#1	TRCN0000073734 5'-CCGGGCCGTAAACTGCTTTGTGAATCTCGAGATTCACAAAGCAGTTTACGGCTTTTTG-3'	Sigma Aldrich
	sh*EPCAM*#2	TRCN0000073737 5'-CCGGCGCGTTATCAACTGGATCCAACTCGAGTTGGATCCAGTTGATAACGCGTTTTTG-3'	Sigma Aldrich
Control non-target shRNA constructs	shNT	SHC016V 5'-CCGGGCGCGATAGCGCTAATAATTTCTCGAGAAATTATTAGCGCTATCGCGCTTTTT-3'	Sigma Aldrich
shRNA-resistant *EPCAM* construct against sh*EPCAM*#1	sh*EPCAM-R*	5'-ATGGCGCCCCCGCAGGTCCTCGCGTTCGGGCTTCTGCTTGCCGCGGCGACGGCGACTTTTGCCGCAGCTCAGGAAGAATGTGTCTGTGAAAACTACAAGCTGGCTGTGAATTGTTTCGTCAACAATAATCGTCAATGCCAGTGTACTTCAGTTGGTGCACAAAATACTGTCATTTGCTCAAAGCTGGCTGCCAAATGTTTGGTGATGAAGGCAGAAATGAATGGCTCAAAACTTGGGAGAAGAGCAAAACCTGAAGGGGCCCTCCAGAACAATGATGGGCTTTATGATCCTGACTGCGATGAGAGCGGGCTCTTTAAGGCCAAGCAGTGCAACGGCACCTCCATGTGCTGGTGTGTGAACACTGCTGGGGTCAGAAGAACAGACAAGGACACTGAAATAACCTGCTCTGAGCGAGTGAGAACCTACTGGATCATCATTGAACTAAAACACAAAGCAAGAGAAAAACCTTATGATAGTAAAAGTTTGCGGACTGCACTTCAGAAGGAGATCACAACGCGTTATCAACTGGATCCAAAATTTATCACGAGTATTTTGTATGAGAATAATGTTATCACTATTGATCTGGTTCAAAATTCTTCTCAAAAAACTCAGAATGATGTGGACATAGCTGATGTGGCTTATTATTTTGAAAAAGATGTTAAAGGTGAATCCTTGTTTCATTCTAAGAAAATGGACCTGACAGTAAATGGGGAACAACTGGATCTGGATCCTGGTCAAACTTTAATTTATTATGTTGATGAAAAAGCACCTGAATTCTCAATGCAGGGTCTAAAAGCTGGTGTTATTGCTGTTATTGTGGTTGTGGTGATAGCAGTTGTTGCTGGAATTGTTGTGCTGGTTATTTCCAGAAAGAAGAGAATGGCAAAGTATGAGAAGGCTGAGATAAAGGAGATGGGTGAGATGCATAGGGAACTCAATGCATAA – 3'	Invitrogen

Primer used to generate the shRNA-resistant *EPCAM* construct		Forward: 5'- aattctgcagtcgacggtaccATGGCGCCCCCGCAGGTC -3'	Eurofins genomics
Primer used to generate the shRNA-resistant *EPCAM* construct		Reverse: 5'- caccatggtggcgaccaggtggatcccgggTGCATTGAGTTCCCTATGCATCTCA –3'	Eurofins genomics
siRNA targeting human ACTN4 mRNA	ACTN4 siRNA#1	5'- CUUCUCUGGUGCCAGAGAA[dT][dT]-3'	Sigma-Aldrich
siRNA targeting human ACTN4 mRNA	ACTN4 siRNA#2	5'- GACAUGUUCAUCGUCCAUA[dT][dT] – 3'	Sigma-Aldrich
siRNA targeting dog *EPCAM* mRNA	*EPCAM* siRNA#1	5'- UUCAUAACCAAACAUUUGGUUGCCA - 3'	Invitrogen
siRNA targeting dog *EPCAM* mRNA	*EPCAM* siRNA#2	5' – UGAUUGAGAGCUGCCUUUCUAUUUA -3'	Invitrogen

Supplementary table 2. **Number of cells (and %) with adhesion and spreading phenotypes, related to Figure 1c.**

	shNT	shEpCAM#1	shEpCAM#2
Attach + / Spread +	100 (99.01%)	42 (34.71%)	51 (45.54%)
Attach + / Spread -	0 (0.00%)	21 (17.36%)	15 (13.39%)
Attach -	1 (0.99%)	58 (47.93%)	46 (41.07%)

Supplementary table 3. **Number of cells (and %) with polarity phenotypes, related to Figure 1i.**

	shNT	shEpCAM#1	shEpCAM#2
Polarized	86 (88.66%)	15 (25.86%)	9 (15.52%)
Unpolarized	10 (10.31%)	25 (43.10%)	28 (48.28%)
C-shape	1 (1.03%)	18 (31.03%)	21 (36.21%)

Supplementary table 4. Number of cells (and %) with polarized or unpolarized phenotype, related to Figure 5e.

	DMSO		Y27632	
	shEpCAM#1	shEpCAM#2	shEpCAM#1	shEpCAM#2
Polarized	3 (1.6%)	2 (1.8%)	49 (24.6%)	63 (30.4%)
Unpolarized	189 (98.4%)	109 (98.2%)	150 (75.4%)	144 (69.6%)

Supplementary Table 5. **Number of focal adhesions (and %) in each size group ($x<1\mu m$, $1<x<2\mu m$, $2<x<3\ \mu m$ and $x>3\mu m$), related to Supplementary Figure 2g.**

	Caco2 shNT	Caco2 shEpCAM#1	Caco2 shEpCAM#2
Length<1μm	9191 (92.6%)	3078 (85.2%)	2857 (78.6%)
1<Length<2μm	650 (6.5%)	406 (11.2%)	572 (15.7%)
2<Length<3μm	76 (0.8%)	101 (2.8%)	158 (4.3%)
Length>3μm	11 (0.1%)	26 (0.7%)	48 (1.3%)
Total	9928 (100%)	3611 (100%)	3635 (100%)
