# Supplementary material for: EpCAM promotes endosomal modulation of the cortical RhoA zone for epithelial organization
Source: Nat Commun. 2021 Apr 13;12:2226. doi: 10.1038/s41467-021-22482-9 (PMC8044225; doi:10.1038/s41467-021-22482-9)
Supplement: Supplementary file 2 — Description of Additional Supplementary Files [file 41467_2021_22482_MOESM2_ESM.pdf]

Descriptions for Additional Supplementary Files

Supplementary Movie 1
Description: 3-hour time lapse imaging of Caco2 shNT cells during spreading and polarity acquisition. Images were acquired every 6 min. Frame rate is 15fps.

Supplementary Movie 2
Description: 3-hour time lapse imaging of Caco2 sh*EPCAM* cells during spreading. Images were acquired every 6 min. Frame rate is 15fps.

Supplementary Movie 3
Description: 4-hour time lapse imaging of Caco2 sh*EPCAM* cells during spreading, showing symmetry breaking events and C-shape acquisition. Images were acquired every 6 min. Frame rate is 15fps.

Supplementary Movie 4
Description: 2-hour time lapse spinning-disc acquisition of Lifeact-GFP dynamics (gray) in Caco2 shNT cells. Images were acquired every 5min. Frame rate is 10fps.

Supplementary Movie 5
Description: 2-hour time lapse spinning-disc acquisition of Lifeact-GFP dynamics (gray) in Caco2 sh*EPCAM* cells. Images were acquired every 5min. Frame rate is 10fps.

Supplementary Movie 6
Description: 2-min time lapse spinning-disc acquisition of AHPH-mCherry dynamics (gray) in Caco2 shNT cells. Images were acquired every 5sec. Frame rate is 15fps.

Supplementary Movie 7
Description: 2-min time lapse spinning-disc acquisition of AHPH-mCherry dynamics (gray) in Caco2 sh*EPCAM* cells. Images were acquired every 5sec. Frame rate is 15fps.

Supplementary Movie 8
Description: 2-min time lapse spinning-disc acquisition of AHPH-mCherry dynamics (gray) in Caco2 shEpCAM-R cells. Images were acquired every 5sec. Frame rate is 15fps.

Supplementary Movie 9
Description: 1-min time lapse spinning-disc acquisition of AHPH-mCherry dynamics (red) together with EHD1-GFP (green) in Caco2 shNT cells. Images were acquired every 1sec. Frame rate is 15fps.

Supplementary Movie 10
Description: Close-up of AHPH-mCherry dynamics (red) together with EHD1-GFP (green) during 15-sec time lapse spinning-disc acquisition in Caco2 shNT cells. Images were acquired every 1sec. Frame rate is 15fps.

Supplementary Movie 11
Description: 1-min time lapse spinning-disc acquisition of AHPH-mCherry dynamics (red) together with EHD1-GFP (green) in Caco2 sh*EPCAM* cells. Images were acquired every 1sec. Frame rate is 15fps.

Supplementary Movie 12

Description: Close-up of AHPH-mCherry dynamics (red) together with EHD1-GFP (green) during 30-sec time lapse spinning-disc acquisition in Caco2 sh*EPCAM* cells. Images were acquired every 1sec. Frame rate is 15fps.
