# Supplementary material for: EpCAM promotes endosomal modulation of the cortical RhoA zone for epithelial organization
Source: Nat Commun. 2021 Apr 13;12:2226. doi: 10.1038/s41467-021-22482-9 (PMC8044225; doi:10.1038/s41467-021-22482-9)
Supplement: Supplementary file 15 — Reporting Summary [file 41467_2021_22482_MOESM15_ESM.pdf]

nature research

Corresponding author(s): DELACOUR Delphine

Last updated by author(s): 2021/03/05

Reporting Summary

Nature Research wishes to improve the reproducibility of the work that we publish. This form provides structure for consistency and transparency in reporting. For further information on Nature Research policies, see our Editorial Policies and the Editorial Policy Checklist.

Statistics

For all statistical analyses, confirm that the following items are present in the figure legend, table legend, main text, or Methods section.

n/a	Confirmed	
☐	☒	The exact sample size (*n*) for each experimental group/condition, given as a discrete number and unit of measurement
☐	☒	A statement on whether measurements were taken from distinct samples or whether the same sample was measured repeatedly
☐	☒	The statistical test(s) used AND whether they are one- or two-sided<br>*Only common tests should be described solely by name; describe more complex techniques in the Methods section.*
☒	☐	A description of all covariates tested
☐	☒	A description of any assumptions or corrections, such as tests of normality and adjustment for multiple comparisons
☐	☒	A full description of the statistical parameters including central tendency (e.g. means) or other basic estimates (e.g. regression coefficient) AND variation (e.g. standard deviation) or associated estimates of uncertainty (e.g. confidence intervals)
☐	☒	For null hypothesis testing, the test statistic (e.g. *F*, *t*, *r*) with confidence intervals, effect sizes, degrees of freedom and *P* value noted<br>*Give P values as exact values whenever suitable.*
☒	☐	For Bayesian analysis, information on the choice of priors and Markov chain Monte Carlo settings
☒	☐	For hierarchical and complex designs, identification of the appropriate level for tests and full reporting of outcomes
☒	☐	Estimates of effect sizes (e.g. Cohen's *d*, Pearson's *r*), indicating how they were calculated

Our web collection on statistics for biologists contains articles on many of the points above.

Software and code

Policy information about availability of computer code

Data collection	Zen Black (11.0.0.190), Biostation IM-Q (2.10), Metamorph (7.10.1.161), Image Lab Touch (2.3.0.07)
Data analysis	Matlab R2018b (9.5.0.94444), Fiji ImageJ (2.0.0-rc-69/1.52p), GraphPad Prism 7 (7.05) and 8 (8.4.1). The code of MATLAB-based custom program for AFM analyses has been published in Staunton et al., Scientific Reports 2016, and is available on GitHub [https://github.com/bryantdoss/matlab-afm-indentation , https://doi.org/10.5281/zenodo.4543281]. The code of MATLAB-based custom program for triple colocalization analyzes was developed for this publication and is available on Github [https://github.com/simondebeco/Colocalization-Analyzer , https://doi.org/10.5281/zenodo.4501944].

For manuscripts utilizing custom algorithms or software that are central to the research but not yet described in published literature, software must be made available to editors and reviewers. We strongly encourage code deposition in a community repository (e.g. GitHub). See the Nature Research guidelines for submitting code & software for further information.

Data

Policy information about availability of data

All manuscripts must include a data availability statement. This statement should provide the following information, where applicable:

- Accession codes, unique identifiers, or web links for publicly available datasets
- A list of figures that have associated raw data
- A description of any restrictions on data availability

The data that support the findings of this study are available from the corresponding author upon request. Raw western blots are already presented in Supplementary Figures 11 and 12.

Field-specific reporting

Please select the one below that is the best fit for your research. If you are not sure, read the appropriate sections before making your selection.

- [x] Life sciences
- [ ] Behavioural & social sciences
- [ ] Ecological, evolutionary & environmental sciences

For a reference copy of the document with all sections, see nature.com/documents/nr-reporting-summary-flat.pdf

Life sciences study design

All studies must disclose on these points even when the disclosure is negative.

Sample size	No statistical methods were used to predetermine sample size. Sample size was determined based on previous studies from our group. We used the same range of data size for the different experimental conditions. We performed at least two to three independent experiments and capture images or movies on multiple cells. In all cases, the sample size used was necessary to get clear and statistically siginificant results. We specified the number of cells analyzed and independent experiments realized in the figure legends and/or in the source data file.
Data exclusions	We did not explicitly excluded any data from our analysis. Figure 1c describes the cell attachment phenotype, so we took into account every cell in this analysis. For all the other dynamic analysis experiments, cells that died and/or became detached during the analysis were not included.
Replication	Statistical analyzed experiments were performed at least 3 times independently. All attempts at replication were successful.
Randomization	Experiments including fluorescence microscopy, cell motility, vesicle tracking, AFM, TFM were analyzed based on randomly selected fields without bias.
Blinding	Due to technical/researcher limitations (the same personnel did the associated experiments) and obvious phenotypes, investigators were not blinded in the data analyses and blinding was not necessary to this study.

Reporting for specific materials, systems and methods

We require information from authors about some types of materials, experimental systems and methods used in many studies. Here, indicate whether each material, system or method listed is relevant to your study. If you are not sure if a list item applies to your research, read the appropriate section before selecting a response.

Materials & experimental systems

n/a	Involved in the study	
☐	☒	Antibodies
☐	☒	Eukaryotic cell lines
☒	☐	Palaeontology and archaeology
☒	☐	Animals and other organisms
☒	☐	Human research participants
☒	☐	Clinical data
☒	☐	Dual use research of concern

Methods

n/a	Involved in the study	
☒	☐	ChIP-seq
☒	☐	Flow cytometry
☒	☐	MRI-based neuroimaging

Antibodies

Antibodies used	anti- EpCAM (abcam, #ab71916, IF dilution, 1:100), anti-zyxin (abcam, #ab58210 (clone denomination not provided by the manufacturer), IF dilution 1:100), anti- paxillin (Merck Millipore, clone 5H11 #05-417, IF dilution, 1:100), anti-talin (Merck Millipore, Clone TA205 #05-385, IF dilution, 1:100), anti-beta1-integrin (BD Biosciences, clone18 #610467, IF dilution: 1/100), anti-vinculin (Sigma-Aldrich, #V4139, IF dilution, 1:100), anti-alpha-tubulin (Sigma-Aldrich, Clone DM1A, #T6199, WB dilution 1:1000), anti-MLC2 (Cell Signaling, #3672, WB dilution 1:1,000), anti-P-MLC2 (Cell Signaling, T18/S19, #3674S IF dilution 1:100, WB dilution 1:500), anti-alpha4-actinin (Invitrogen, #42-1400 (clone denomination not provided by the manufacturer), IF dilution, 1:100), anti-GAPDH (Proteintech, #60004-1-Ig, clone 1E6D9, WB dilution, 1:500), anti-Myosin-IIA (Biolegend, #909801, IF dilution 1:100), anti-EEA1 (BD, #610457, clone 14/EEA1, IF dilution 1:100), anti-LAMP1 (BD, #555798, Clone H4A3, IF dilution 1:100), HRP-linked anti-mouse IgG (Sigma-Aldrich, #A9044 , dilution 1:10,000), HRP-linked anti-rabbit IgG (GE Healthcare, #NA934-1ML, dilution 1:10,000), Alexa-488 anti-mouse (Invitrogen, #A-11001, dilution 1:250), Alexa-488 anti-rat (Invitrogen, #A-11006, dilution 1:250), Alexa-488 anti-rabbit (Invitrogen, #A-11008, dilution 1:250), Alexa-568 anti-mouse (Invitrogen, #A-11004, dilution 1:250), Alexa-568 anti-rabbit (Invitrogen, #A-11011, dilution 1:250).
Validation	EpCAM ab: ab71916 has been referenced in 67 publications according to the manufacturer. ab71916 is notably suitable for Western blot and immunofluorescence, and reacts with human, mouse, rat and dog based on the manufacturer website.<br>Zyxin ab : ab58210 has been referenced in 2 publications according to the manufacturer. ab58210 is notably suitable for immunofluorescence, and reacts with human and mouse based on the manufacturer website.<br>Paxillin ab : 05-417 has been referenced in 17 publications according to the manufacturer. 05-417 is notably suitable for

immunofluorescence and reacts with human, mouse and rat based on the manufacturer website.
LAMP1 Clone H4A3 antibody has been referenced in 12 publications according to the manufacturer. Clone H4A3 is notably suitable for immunofluorescence and reacts with human according to the manufacturer website.
EEA1 #610457 antibody has been referenced in 5 publications according to the manufacturer. #610457 is notably suitable for immunofluorescence and reacts with human, dog and chicken according to the manufacturer website.
Talin ab : 05-385 has been referenced in 5 publications according to the manufacturer. 05-385 is notably suitable for immunohistochemistry and reacts with human, mouse and rabbit according to the manufacturer website.
anti-beta1-integrin ab : 610467 has been referenced in 5 publications according to the manufacturer. 610467 is notably suitable for immunofluorescence and reacts with human, mouse, rat and dog according to the manufacturer website.
Vinculin ab : V4139 has been referenced in 51 publications according to the manufacturer. V4139 is notably suitable for immunofluorescence and reacts with human, mouse and rat according to the manufacturer website.
Alpha-tubulin ab : T6199 has been referenced in 1286 publications according to the manufacturer. T6199 is notably suitable for Western blot and reacts with human, mouse and rat according to the manufacturer website.
MLC2 ab : 3672 has been referenced in 177 publications according to the manufacturer. 3672 is notably suitable for Western blot and reacts with human, mouse and rat according to the manufacturer website.
P-MLC2 ab : 3674S has been referenced in 222 publications according to the manufacturer. 3674S is notably suitable for Western blot and immunofluorescence, and reacts with human and mouse according to the manufacturer website.
alpha4-actinin ab : 42-1400 has been referenced in 6 publications according to the manufacturer. 42-1400 is notably suitable for Western blot, immunofluorescence and immuniprecipitation, and reacts with human, mouse and rat according to the manufacturer website.
GAPDH ab : 60004-1-Ig has been referenced in 2088 publications according to the manufacturer. 60004-1-Ig is notably suitable for Western blot and reacts with human, mouse and rat according to the manufacturer website.
Myosin-IIA ab : 909801 has been referenced in 29 publications according to the manufacturer. 909801 is notably suitable for Western blot and immunocytochemistry, and reacts with human, mouse and rat according to the manufacturer website.

Eukaryotic cell lines

Policy information about cell lines

Cell line source(s)	Although Caco2, MDCK, U2OS and HeLa cells were kindly provided by collaborators, they were initially acquired from ATCC.
Authentication	Cell lines were authenticated based on their morphology. Only cultures of low passages were used.
Mycoplasma contamination	All cell lines were mycoplasma-tested and negative.
Commonly misidentified lines (See ICLAC register)	None
